# Exploiting the fibroblast growth factor receptor-1 vulnerability to therapeutically restrict the MYC-EZH2-CDKN1C axis-driven proliferation in Mantle cell lymphoma

**DOI:** 10.1038/s41375-023-02006-8

**Published:** 2023-08-19

**Authors:** Anuvrat Sircar, Satishkumar Singh, Zijun Y. Xu-Monette, Krysta Mila Coyle, Laura K. Hilton, Evangelia Chavdoula, Parvathi Ranganathan, Neeraj Jain, Walter Hanel, Philip Tsichlis, Lapo Alinari, Blake R. Peterson, Jianguo Tao, Natarajan Muthusamy, Robert Baiocchi, Narendranath Epperla, Ken H. Young, Ryan Morin, Lalit Sehgal

**Affiliations:** 1 Division of Hematology, College of Medicine, The Ohio State University, Columbus, OH USA; 2 The Ohio State University Comprehensive Cancer Center-Arthur G. James Cancer Hospital and Richard J. Solove Research Institute, Columbus, OH USA; 3 Division of Hematopathology, Department of Pathology, Duke University Medical Center, Durham, NC USA; 4 Department of Molecular Biology & Biochemistry, Simon Fraser University, Burnaby, BC Canada; 5 Centre for Lymphoid Cancer, British Columbia Cancer, Vancouver, BC Canada; 6 Department of Cancer Biology and Genetics, The Ohio State University, Columbus, OH USA; 7 Division of Cancer Biology, CSIR-Central Drug Research Institute, Lucknow, Uttar Pradesh 226031 India; 8 Academy of Scientific and Innovative Research, Ghaziabad, Uttar Pradesh 201002 India; 9 Division of Medicinal Chemistry and Pharmacognosy, College of Pharmacy, The Ohio State University, Columbus, OH USA; 10 Division of Pathology, University of Virginia, Charlottesville, VA USA; 11 Duke Cancer Institute, Durham, NC USA; 12 Canada’s Michael Smith Genome Sciences Centre, British Columbia Cancer, Vancouver, BC Canada

**Keywords:** B-cell lymphoma, Targeted therapies

## Abstract

Mantle cell lymphoma (MCL) is a lethal hematological malignancy with a median survival of 4 years. Its lethality is mainly attributed to a limited understanding of clinical tumor progression and resistance to current therapeutic regimes. Intrinsic, prolonged drug treatment and tumor-microenvironment (TME) facilitated factors impart pro-tumorigenic and drug-insensitivity properties to MCL cells. Hence, elucidating neoteric pharmacotherapeutic molecular targets involved in MCL progression utilizing a global “unified” analysis for improved disease prevention is an earnest need. Using integrated transcriptomic analyses in MCL patients, we identified a Fibroblast Growth Factor Receptor-1 (FGFR1), and analyses of MCL patient samples showed that high FGFR1 expression was associated with shorter overall survival in MCL patient cohorts. Functional studies using pharmacological intervention and loss of function identify a novel MYC-EZH2-CDKN1C axis-driven proliferation in MCL. Further, pharmacological targeting with erdafitinib, a selective small molecule targeting FGFRs, induced cell-cycle arrest and cell death in-vitro, inhibited tumor progression, and improved overall survival in-vivo. We performed extensive pre-clinical assessments in multiple in-vivo model systems to confirm the therapeutic potential of erdafitinib in MCL and demonstrated FGFR1 as a viable therapeutic target in MCL.

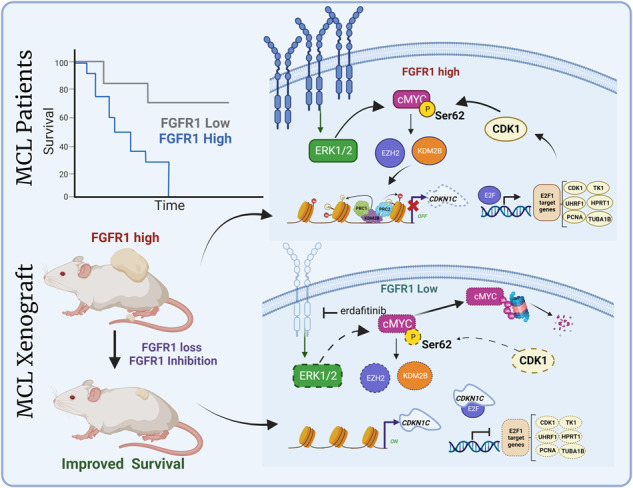

## Introduction

Mantle cell lymphoma (MCL) is an aggressive mature B-cell neoplasm [1, 2], with 60% of patients showing bone marrow involvement at diagnosis [3, 4]. Despite intensive therapeutic approaches, the median progression-free survival after first-line treatment is four years [5–7]. The emergence of chemoresistance is rapid, durable responses to second and third-line therapies are rare, and relapse is virtually universal in MCL [8–10]. However, for most patients who progress on targeted agents like ibrutinib, survival is only 6-10 months [8, 9, 11]. The interaction of MCL with the tumor microenvironment (TME) provides specific niches for lymphoma cells to communicate and promote growth and resistance to chemotherapeutic agents [12]. Therefore, the identification of clinically relevant targets and the development of new therapies are urgently needed for MCL patients.

Cell cycle dysregulation is a hallmark of MCL, resulting from a t(11;14) chromosomal translocation that drives unrestrained proliferation of cancer cells [13, 14]. While the Eμ-Cyclin D1 mouse model does not develop MCL [15], Sox11 and CyclinD1 are essential MCL biomarkers and upregulated in 80% of MCL cases. Eµ-SOX11:CCND1 double transgenic (DT) mice exhibit cell cycle programming and significant enrichment of E2F1 target genes [16]. Rb, a tumor suppressor protein often mutated in MCL, binds to and blocks the activating E2F transcription factors [17, 18]. CDK4/6 may form a complex with cyclin D1 to phosphorylate Rb [19], resulting in E2F transactivation leading to transition of G1 cells to the S phase [19]. However, clinical trials of CDK inhibitors in MCL, such as Palbociclib [20] (CDK4/6 inhibitor), showed an overall response rate (ORR) of 18% and complete response (CR) of 8% in previously treated MCL [20].

Fibroblast growth factor receptors (FGFRs), transmembrane receptor tyrosine kinases, play an essential role in development, differentiation, cell survival, migration, angiogenesis, and carcinogenesis [21–30]. The FGFR pathway is the third most frequently altered pathway in cancer, with the most common abnormality being *FGFR1* amplification [31]. Fusion of the B-cell receptor with FGFR1, utilizing the kinase activity of FGFR1, has been reported in ALL but not in MCL [32–34]. Clinical trials of CDK inhibitors in MCL that block RB phosphorylation to regulate E2F1 mediated transactivation, such as palbociclib [20] (CDK4/6 inhibitor), showed promise alone or in combination with ibrutinib in relapsed MCL [20]. Interestingly, FGFR1 signaling is shown to mediate resistance to Palbociclib in other disease models [35].

In summary, we identified a novel role of FGFR1 in MCL survival, regulating cell cycle-dependent processes primarily by activating E2F1-mediated transactivation through epigenetic repression of CDKN1C. We show that high FGFR1 expression is associated with a poor prognosis in MCL patients. Functionally, genetic knockdown of FGFR1 or pharmacological targeting with erdafitinib, a selective small molecule targeting FGFRs, induced cell cycle arrest, cell death in-vitro, reduced tumor formation, and improved overall survival in-vivo. Our findings point toward the FGFR1 signaling pathway as a critical modulator of cell survival and represent a novel and promising candidate for targeted therapy for patients with relapsed MCL.

## Materials and Methods

### Patient samples

Peripheral blood from healthy donors and peripheral blood, bone marrow samples, and lymph nodes from MCL patients were obtained from Leukemia Tissue Bank, OSU, following written informed consent under a protocol approved by the Institutional Review Board of OSU per the Declaration of Helsinki. MCL PDX cells were obtained from PROxe and were used for ex-vivo analysis.

*Further detailed information on experimental procedures, methods, and materials is available in the Supplementary file*.

## Results

### Elevated FGFR1 is associated with poor survival in MCL patients

**Fig. 1:**
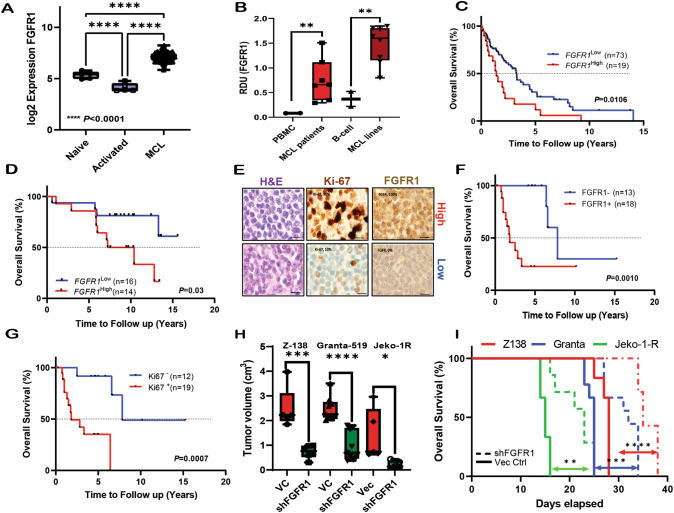
Elevated FGFR1 is associated with poor survival in MCL Patients, and loss of FGFR1 reduces tumorigenicity in-vivo. **A** *FGFR1* expression is elevated in MCL patients (n = 122) compared to naïve or activated B-cells. *Ordinary one-way ANOVA*. **B** Expression of FGFR1 protein is elevated in MCL primary MCL (n = 4) three MCL PDX compared to two healthy donor Peripheral blood mononuclear cells (PBMC). Expression of FGFR1 protein is elevated in MCL cell lines compared to healthy donor CD19 + B-cells. *Unpaired T-test*. **C** Rosenwald cohort (LLMP) was analyzed for *FGFR1* expression in MCL Patients (n = 92), and the Kaplan Meier plot for overall survival was generated and analyzed with the Log-rank Mantel-Cox test. **D** Morin Cohort was analyzed for *FGFR1* expression MCL Patients who received CHOP/R (≤6 cycles) (n = 30), and Kaplan Meier plots for overall survival were generated and analyzed with the Log-rank Mantel-Cox test. **E** Representative images of Hematoxylin-eosin (H&E) staining and immunohistochemical (IHC) staining of Ki-67 and FGFR1 in MCL patient tumor samples from Young Cohort (n = 31). **F** FGFR1 or (**G**) Ki67 expression in MCL Patients (Young Cohort; n = 31) accessed by hematopathologists and Kaplan Meier plots for overall survival were generated and analyzed with the Log-rank Mantel-Cox test. **H** Tumor volume in NSG mice injected with FGFR1 VC vs. knockdown Z-138 (VC n = 5, shFGFR1 n = 6), Granta-519 (VC n = 9, shFGFR1 n = 9), and Jeko-1R (VC n = 5, shFGFR1 n = 5) cells, measured at ERC of first VC mouse. *Unpaired T-test* (**I**) Kaplan-Meier graphs to show the overall survival of NSG mice injected with either FGFR1 VC or knockdown Z-138, Granta-519, and Jeko-1R cells. p-values were calculated by the Log-rank Mantel-Cox test.

Previously it has been shown that MCL–TME physical interactions with the stromal cells confer MCL survival and drug resistance [36]. To confirm whether physical cell contact was indispensable for MCL cell growth, we cultured patient-derived MCL cell lines in the presence or absence of stromal-conditioned media (CM) and investigated the impact on MCL cell proliferation and drug resistance. As shown in Fig. S1A, the culture of MCL cell lines, MCL PDX ex-vivo, and mouse MCL cells with bone marrow (BM) stromal CM (HS-5CM) significantly increased growth and abrogated response to drugs (doxorubicin, ibrutinib, and acalabrutinib) (Fig. S1B-C). To identify critical up-regulated genes driving MCL proliferation, bulk transcriptome analyses on RNA-seq data (p < 0.01; FC > 1.6) from (a) MCL patient vs. healthy-donor, (b) MCL ibrutinib-relapsed vs. treatment-naïve patients, (c) MCL with/without BM, and, (d) a chronic ibrutinib resistance MCL model described previously [36], were overlapped to identify up-regulated genes. The integrated analysis identified four genes (*FGFR1, ABRACL, IFI27, and GEN*) (Fig. S1D; Supplementary Table 1-2). Fibroblast Growth Factor Receptor-1 (*FGFR1*) showed minimal variation (coefficient of variation<6%) among MCL patients (n = 122) (Fig. S1E). However, *FGFR1* is overexpressed at the transcript level in MCL Patients (n = 122) compared to naïve or activated-B cells from healthy donors (Fig. 1A) as analyzed in publicly available datasets (details in supplementary methods). In addition, FGFR1 levels were higher in MCL primary patient samples and MCL cell lines compared to healthy donor PBMC or B cells (Fig. 1B), primary MCL or MCL cell lines cultured with HS5CM, and resistant MCL cell lines described previously [36] (Fig. S1F). High *FGFR1* expression in Rosenwald Cohort [37] treated with multiagent therapy(n = 92) or in Morin Cohort treated with R/CHOP (n = 30) show significant adverse effects on overall survival (OS) (Fig. 1C-D; Supplementary Table 3). Immunohistochemical staining of FGFR1 and Ki67 in MCL patients (n = 31) (Our cohort; Supplementary Table 3) uniformly treated with front-line R/CHOP and normal lymph node controls were performed and analyzed by a hematopathologist (Fig. 1E) for validation. Using the X-tile approach, the cut-off for FGFR1 expression (FGFR1^+^) was set at ≥10%; 18 (58%) of the 31 patients had high expression (FGFR1^+^), and 13 (42%) had low levels of FGFR1(FGFR1^-^). Retrospective analysis of MCL patients with FGFR1 expression showed significantly poorer OS (p = 0.001; Fig. 1F) and high Ki67 expression (61%) in MCL patients correlated with poor OS (p = 0.0007; Fig. 1G). Analyses of staining for FGFR1 and Ki67 in MCL patients revealed that MCL patient tumors with high FGFR1 expression significantly expressed higher Ki67 (Fig. S2A). To understand whether FGFR1’s prognostic effects depended on Ki67, we integrated FGFR1 and Ki67 protein data. In Ki67^+^ cases, FGFR1 continued to show significant adverse effects on overall survival (OS) with median overall survival of 1.75 years, compared to 6.4 years in Ki67^+^ FGFR1^-^ patients (Fig. S2B). Further validation in Morin cohort [38] shows that *MKI67* was associated with significant adverse prognostic effects (Fig. S2C), and in MKI67^High^ cases, FGFR1 continued to show adverse effects on overall survival (OS) (Fig. S2D). Genomic analysis of the available 26 patients in the Morin cohort did not show any mutation in *FGFR1* (supplementary table 3). Moreover, an extension of our analysis to other available studies [38, 39] in MCL (n = 162) did not reveal any activating mutation in the *FGFR1 gene* (Fig. S2E). Proliferation gene signature (PSG) in MCL is an average of the proliferative genes defined by Rosenwald, and we found that both FGFR1 and PSG continued to show significant adverse effects on overall survival in MCL Patients (n = 92) (Fig. 1C and Fig. S2F). Moreover, PSG^High^FGFR1^High^ MCL Patients show significant adverse effects on overall survival (OS) with median overall survival of 0.89 years, compared to 2.2 years in PSG^High^FGFR1^Low^ patients (Fig. S2G). Our findings validated in multiple patient cohorts suggest that FGFR1 has independent adverse prognostic effects on overall survival in MCL patients.

### Loss of FGFR1 reduces tumorigenicity in-vitro and in-vivo

To test if FGFR1 contributes to MCL’s aggressive cell growth and survival, we generated knockdown clones in MCL cell lines (Z-138, Granta-519, Jeko-1R, and SP49-R) using different shRNA constructs (Fig. S2H-I). Next, to determine the role of FGFR1 in neoplastic progression in-vivo, we injected vector control (Vec Ctrl; VC) or the shFGFR1 clones of three representative MCL cell lines (Z-138, Granta-519, and Jeko-1R) into the flanks of NSG mice and measured tumor volume until the ERC of any experimental mice was reached. Consistent with our in-vitro studies, mice transplanted with shFGFR1 clones showed reduced tumor volume compared to vector controls (Fig. 1H) and exhibited a significantly prolonged median overall survival compared to control mice (shFGFR1 vs. Vec: Z138 [35 days vs. 28 days]; Granta-519 [32 days vs. 25]; Jeko-1-R [23 days vs. 15 days]) (Fig. 1I). However, despite this improvement, the engrafted mice eventually reached ERC (end-point of the study), in addition, the survival advantage for Jeko-1R was modestly significant. Our results demonstrate that FGFR1 is up-regulated in MCL, and depletion of FGFR1 alleviates tumor burden and improves survival in-vivo in MCL cell-derived xenograft models.

### FGFR1 can be therapeutically targeted using a selective inhibitor of FGFRs

**Fig. 2:**
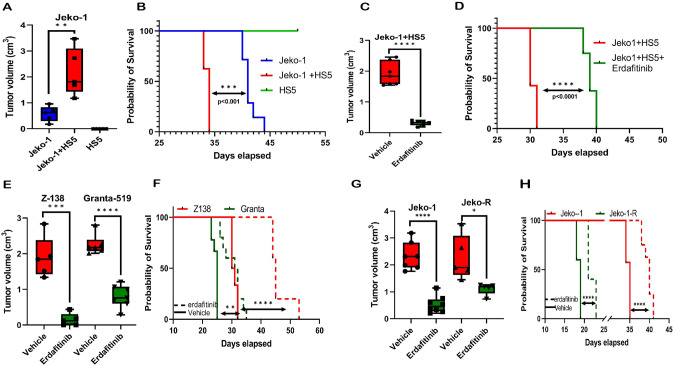
FGFR1 can be therapeutically targeted using the selective inhibitor erdafitinib. **A** Tumor volume at ERC of the first mouse was measured when Jeko-1 cells alone (n = 5) or combined with HS-5 cells (n = 5) were injected subcutaneously in NSG mice and subsequently treated with erdafitinib. HS-5 cells alone (n = 5) were injected in a separate group of mice as a control. **B** The Kaplan-Meier graph shows the survival time for mice described in (**A**). **C** Tumor volume at ERC of the first mouse was measured when NSG mice were injected subcutaneously with Jeko-1 cells in combination with HS-5 cells and subsequently treated with vehicle (n = 7) or erdafitinib (n = 7). **D** The Kaplan-Meier graph shows the survival time for mice described in (**C**). **E** Tumor volume at ERC of the first mouse was measured when NSG mice were injected subcutaneously with Z-138 and Granta-519 and subsequently treated with vehicle (Z138 n = 5, Granta-519 n = 6) or erdafitinib (Z138 n = 5, Granta-519 n = 7). **F** The Kaplan-Meier graph shows the survival time for mice described in (**E**). **G** Tumor volume at ERC of the first mouse was measured when NSG mice were injected subcutaneously with Jeko-1 and Jeko-1R and subsequently treated with vehicle (Jeko-1 n = 7, Jeko-1R n = 5) or erdafitinib (Jeko-1 n = 8, Jeko-1R n = 5). **H** The Kaplan-Meier graph shows the survival time for mice described in (**G**). *Unpaired T-tests* were used for tumor volume analysis, and p-values for survival analysis were calculated using the *Log-rank (Mantel-Cox) test*.

To explore the therapeutic potential of targeting FGFR1 in MCL, we first confirmed the expression of other FGFR family members (*FGFR2-4*) in MCL patients. Notably, other FGFR homologs (*FGFR2-4*) were not significantly up-regulated in MCL as compared to B cells (Fig. S3A-C), and *FGFR1* was the only FGFR homolog significantly up-regulated in both MCL primary patient samples and cell lines (Fig. S3D-E). We could not detect the expression of *FGFR2* using either Taqman or SYBR green Real-time PCR in MCL cell lines. Erdafitinib (JNJ-42756493) is an oral selective pan-FGFR kinase inhibitor [40, 41], with potent antitumor activity in pre-clinical studies exhibiting limited off-target effects on other kinases [40]. In-vitro anti-MCL activity of erdafitinib was demonstrated in MCL cell lines, which showed dose-dependent cellular death as determined by annexin-PI staining (Fig. S3F); further addition of HS5-CM failed to promote the proliferation of MCL cells in the presence of erdafitinib in-vitro (Fig. S3G-H). Since our in-vitro data suggested adding HS5-CM to MCL cells up-regulated FGFR1, we questioned a) whether stroma can promote MCL tumor progression in-vivo and b) if treatment with erdafitinib can abrogate stromal-mediated tumor progression in-vivo. To answer this, we utilized cell-line-derived xenograft (CDX) models of MCL cells (Jeko-1) injected subcutaneously in NSG mice with a) Jeko-1 cells alone, b) HS5 cells alone, and c) Jeko-1 and HS5 cells together, and measured tumor volume until the ERC of any experimental mice was reached. No tumor formation was observed in HS5 cells alone, confirming that normal human stromal cells do not exhibit neoplastic properties in-vivo. However, Jeko-1 cells injected with HS5 cells showed significantly higher tumor volume (p < 0.001; Fig. 2A) and significantly shorter overall survival (34 days) compared to control (41 days; Fig. 2B), suggesting the involvement of stromal cells in facilitating the growth of Jeko-1 cells in-vivo. Conversely, treatment with erdafitinib increased the overall survival of Jeko-1 cells injected subcutaneously in NSG mice with HS5 cells, and these mice had a statistically significant decrease in tumor volume (Fig. 2C-D). Next, the representative MCL cell lines Z-138, Granta-519, and Jeko-1 cells were independently injected subcutaneously into NSG mice flanks, followed by subsequent treatment with erdafitinib. We found erdafitinib treatment to limit tumor progression significantly (p < 0.001) and increase the overall survival in-vivo (p < 0.001; Fig. 2E-H). Next, we found that Jeko-1R cells reached ERC faster than Jeko-1 cells when implanted subcutaneously in NSG mice and that Jeko-1R have increased tumor volume compared to Jeko-1 injected mice (Fig. S3I-J). Furthermore, the tumor volume of the mice implanted with Jeko-1R treated with erdafitinib showed a modest reduction in tumor volume (Fig. 2G) and improved overall survival (Fig. 2H). Our results demonstrate that erdafitinib treatment in MCL alleviates tumor burden and improves survival in-vivo in MCL cell-derived xenograft models.

### Loss of FGFR1 affects cell cycle progression and downregulates the expression of EZH2

**Fig. 3:**
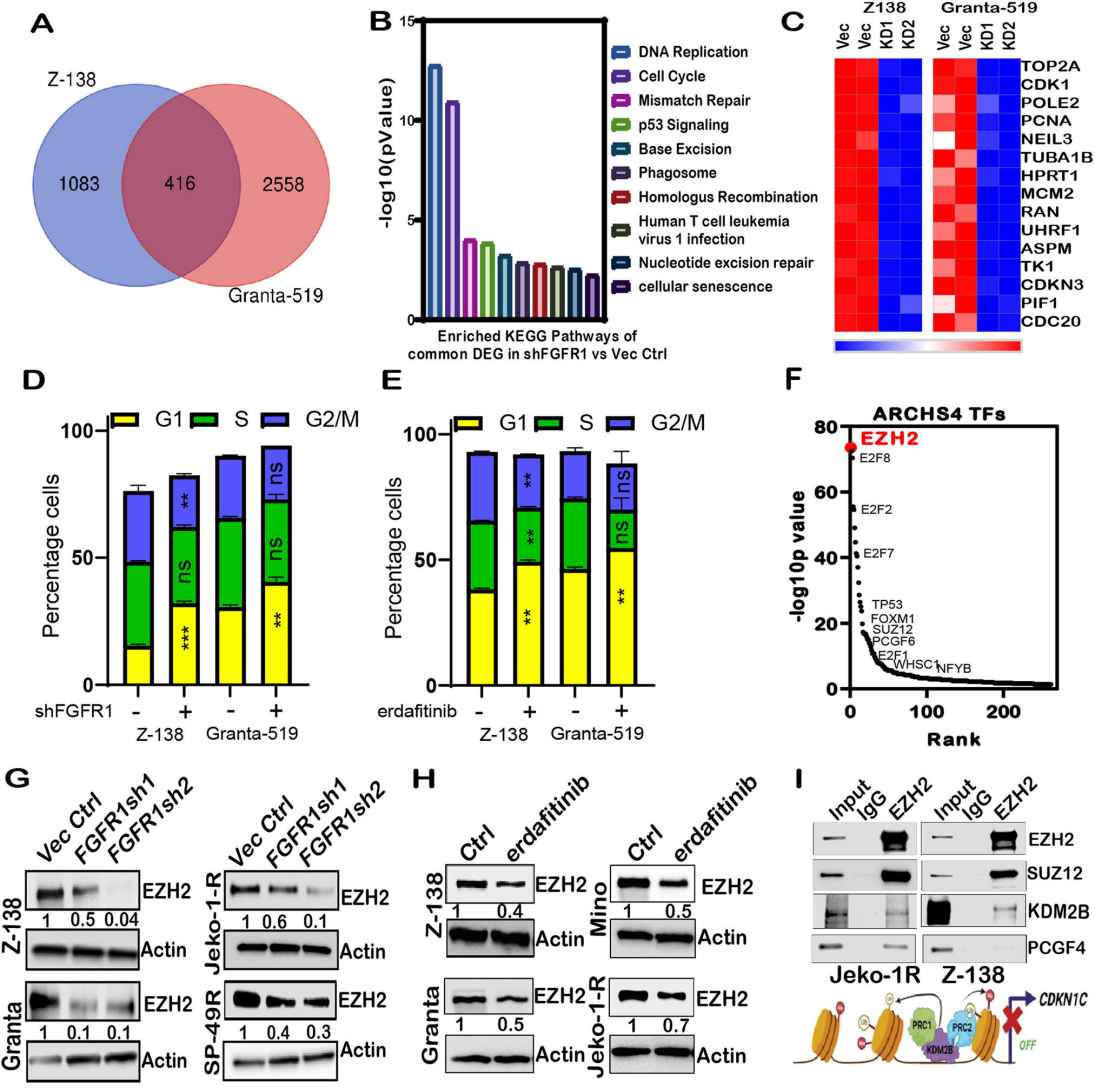
Loss of FGFR1 affects cell cycle progression and downregulates the expression of PRC2 core member EZH2. (**A**) Venn Diagram showing the differentially expressed gene in shFGFR1 vs. Vec ctrl analysis (p < 0.05, FC > 2) (**B**) Enrichment of the DEG obtained in panel A using Enrichr (**C**) Heatmap to show expression of MCL proliferation signature genes (PSG) in FGFR1 VC vs. knockdown cells of MCL cell lines Z-138 and Granta-519. (D) Cell cycle analysis determined the percentage of the G1 population in VC vs. shFGFR1 cells of Z-138 and Granta-519. (**E**) Cell cycle analysis determined the percentage of the G1 population in control vs. erdafitinib treated (16 h) cells of Z-138 and Granta-519. *2-way ANOVA (a* = *0.05)*. (**F**) The hockey plot for the TF co-expressed with DEG was determined by ARCHS analysis. (**G**) Western blots show EZH2 expression in Z-138, Granta-519, Jeko-1R, and SP-49R control vs. shFGFR1 cells; EZH2 expression in Z138 was probed on the same blot from Fig. S2H. (**H**) Western blots show EZH2 expression in Z-138, Granta-519, Mino, and Jeko-1R cells upon erdafitinib treatment. (**I**) Co-immunoprecipitation of EZH2 and KDM2B (PRC1.1), SUZ12 (PRC2) or PCGF4 (PRC1) followed by western blot in Jeko-1R and Z-138 cells. 2% input was loaded with IP with appropriate IgG isotype control, followed by an IP-EZH2 sample.

Next, we performed gene expression profiling with knockdown of FGFR1 in two representative MCL cell lines (Z-138 and Granta-519) to identify common differentially expressed genes (DEG) (Fig. 3A; Supplementary Table 4). Further, KEGG 2021 human pathway analysis on DEG using enricher [42] identified the cell cycle and DNA replication as significantly altered pathways (Fig. 3B; Supplementary Table 5). Cell cycle dysregulation [14] and a defined PSG are MCL hallmarks [37, 43]. Furthermore, we found that FGFR1 loss in MCL down-regulates the expression of genes included in PSG (Fig. 3C) and show a higher number of cells in the G1 phase in shFGFR1 and erdafitinib MCL cells (Fig. 3D-E). Next, enricher-enabled ARCHS analysis [44] revealed EZH2, a histone methyl transferase and core member of the PRC2 complex, to be the most significant transcription factor co-expressed with DEG (Fig. 3F; Supplementary Table 6). Previously we have shown that EZH2 expression is up-regulated in MCL and is associated with poor prognosis [45]; hence we asked if FGFR1 regulates the expression of EZH2. We found EZH2 downregulated at both the RNA (Fig. S4A) and protein levels upon FGFR1 knockdown (Fig. 3G) or treatment with erdafitinib (Fig. 3H). Moreover, inhibiting EZH2 activity using the EZH2 inhibitor tazemetostat or with EZH1/2 dual inhibitor valemetostat increased the percentage of cells in the G1-phase (Fig. S4B). Notably, the expression of SUZ12 (Fig. S4C-D) and no other PRC1/2 members tested were consistently altered upon FGFR inhibition in MCL cell lines. Next, we confirmed that EZH2 forms a complex in MCL cells with PRC2(SUZ12)-PRC1(PCGF4) and PRC1.1 protein(KDM2B) (Fig. 3I). Moreover, we also found the expression of KDM2B to be downregulated in shFGFR1 MCL cells (Fig. S4E), and ectopic expression of KDM2B rescued the expression of EZH2 (Fig. S4F), confirming FGFR1 mediated regulation of KDM2B and EZH2 in MCL.

### EZH2 binds to the noncanonical PRC1.1 KDM2B and regulates the expression of the Cip/Kip2 protein CDKN1C

**Fig. 4:**
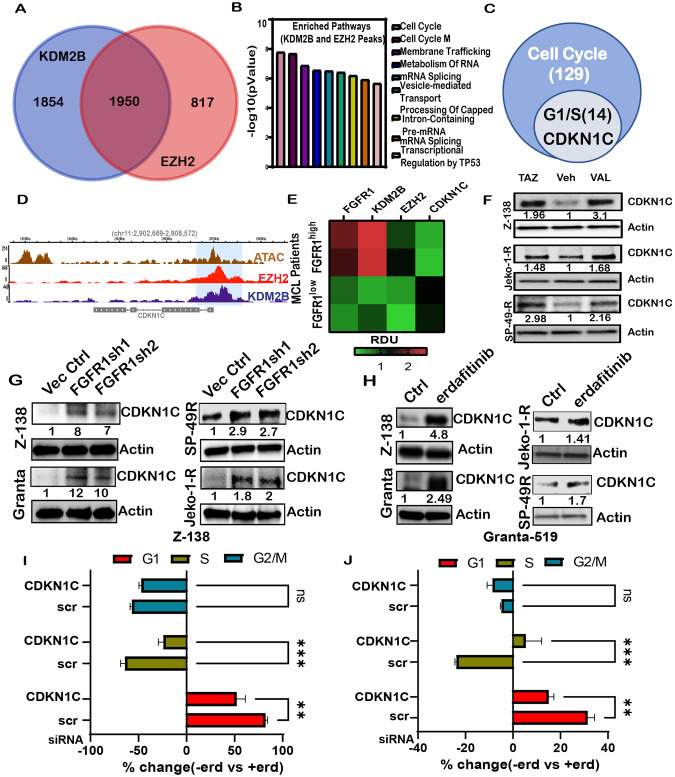
EZH2 and KDM2B co-occupies and negatively regulate the expression of Cip/Kip2 protein CDKN1C. **A** Co-occupied peaks of KDM2B and EZH2 in Z138 cells (within 2KB of TSS. **B** Enriched pathways analysis using Enrichr for the co-occupied 1950 peaks from panel A showing enrichment of cell cycle pathways. **C** Cell cycle-related loci (129) were functionally distinguished using enricher to identify 14 genes with functional relation to G1/S transition, including CDKN1C. **D** Genomic browser (hg19) for enrichment KDM2B and EZH2 on the target CDKN1C Loci with an overlay of ATAC in Z138 cells. **E** Heatmap showing Relative Densitometric Unit (RDU) calculated from Western Blot expression of FGFR1, KDM2B, EZH2, and CDKN1C in FGFR1 high vs. low MCL patient samples. **F** Western blot depicting CDKN1C increase in expression upon tazemetostat and valemetostat treatment in Z-138, Jeko-1R, and SP-49R cells. *Veh: vehicle control*. **G** Western blots to show CDKN1C expression in Z-138, Granta-519, Jeko-1R, and SP-49R control vs. shFGFR1 cells. **H** Western blots to show EZH2 expression in Z-138, Granta-519, Jeko-1R, and SP-49R cells upon erdafitinib treatment. **I** Percent increase in G1 cell percentage in Z-138 and (**J**) Granta-519 cells upon transfection with scrambled si-RNA (control) and si-CDKN1C and subsequent treatment with erdafitinib. *2-way ANOVA (a* = *0.05)*.

To identify genomic regions co-occupied by EZH2 and KDM2B, we performed Cut and Run chromatin immunoprecipitation (ChIP) followed by Next-gen sequencing using validated antibodies (Supplementary Table 7). As shown in Fig. S5A, EZH2, and KDM2B bind primarily to promoter regions. Overlay of both KDM2B and EZH2 peaks identified 1950 common peaks in the promoter regions ( ± 2KBTSS) (p < 0.0001) in MCL (Fig. 4A). Further enrichment analysis on the co-occupied peaks showed enrichment in cell cycle pathways (Fig. 4B; Supplementary Table 8), of which 14 peaks pertain to the promoters of genes involved in G1 to S cell cycle control (Fig. 4C), including a cell-cycle dependent kinase inhibitor 1C (CDKN1C) (Fig. 4D). In addition, we found that MCL patients that express high FGFR1 protein levels have higher expression of both KDM2B and EZH2 and lower levels of CDKN1C, as opposed to MCL patients expressing low FGFR1 (Fig. 4E). This observation, combined with the co-occupied binding of KDM2B and EZH2 on CDKN1C, led us to hypothesize that FGFR1 negatively regulates CDKN1C, primarily through epigenetic repression. Next, using ChIP with an H3K27me3-validated antibody followed by qRT-PCR, we show a repressive mark on the promoter of CDKN1C (Fig. S5B). Further, treatment of MCL cells with either tazemetostat or valemetostat restored CDKN1C expression(Fig. 4F). Since there are no specific KDM2B inhibitors, we performed transient knockdown of both KDM2B and EZH2 and found restoration of CDKN1C (Fig. S5C), similar to inhibition of EZH2, confirming a role of KDM2B and EZH2 to repress the expression of CDKN1C in MCL cells. Since FGFR1 loss downregulates the expression of both KDM2B and EZH2, we asked a) if loss or inhibition of FGFR1 in MCL can restore the expression of CDKN1C and b) if restoration of CDKN1C is needed for a high percentage of G1 Phase cells in MCL. Compared to controls, we found upregulation of CDKN1C protein expression in shRNA FGFR1 MCL cells (Fig. 4G) and upon treatment with erdafitinib (Fig. 4H). Further, erdafitinib treatment in MCL cells shows a higher percentage of cells in the G1-Phase, similar to shFGFR1 clones(Fig. 3D-E). To determine if the observed increase in G1 percentage in MCL cells is due to the restoration of CDKN1C, we knocked down CDKN1C (siRNA) in the presence of erdafitinib; as expected, CDKN1C loss significantly reduced the percent change of G1 cells in response to treatment (Fig. 4I, J). This role of CDKN1C in regulating G1 arrest in MCL agrees with previously published studies [46–48] in other disease models, strengthening our observation.

### CDKN1C binds to E2F1 and regulates the transactivation of its target genes

**Fig. 5:**
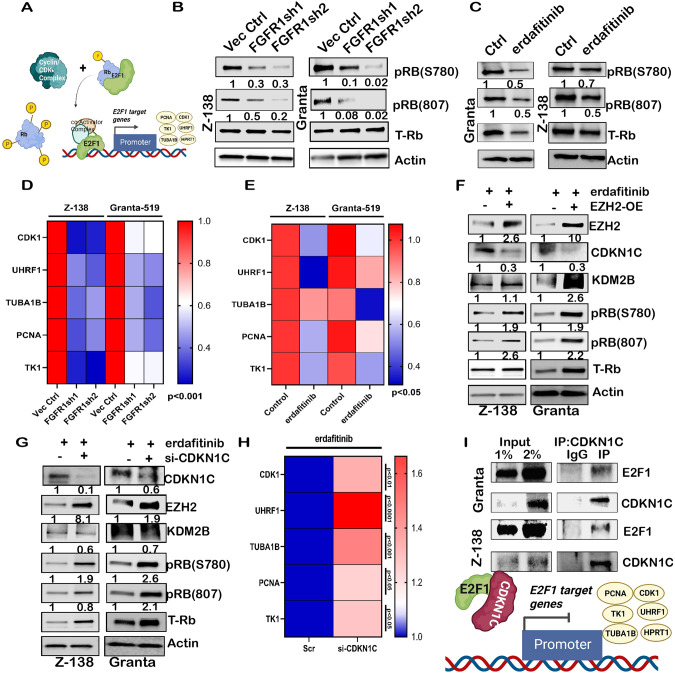
CDKN1C upregulation halts the transactivation of E2F1 target genes in FGFR1 dependent manner. **A** Model showing Rb-E2F1 regulation by Cyclin-CDK complex. **B** Blots show decreased Rb phosphorylation upon FGFR1 knockdown in Z-138 and Granta-519 cell lines. (C) Blots show a decreased Rb phosphorylation upon erdafitinib treatment in Z-138 and Granta-519 cell lines, Actin for Z-138 and Granta-519 in (**B**) is the same as Fig. 3G, and in (**C**) is the same as Fig. 3H (different exposures), as the same blots were probed for Rb and EZH2 (**D**) Heatmap for qPCR analysis depicting the decrease in expression of E2F1 target genes upon FGFR1 knockdown in Z-138 and Granta-519 cell lines. **E** Heatmap for qPCR analysis depicts decreased expression of E2F1 target genes upon erdafitinib treatment in Z-138 and Granta-519 cell lines. *2-way ANOVA (a* = *0.05)*. **F** Blots of the rescue of EZH2 expression upon transfection of EZH2 overexpression plasmid in the presence of erdafitinib and the corresponding change in CDKN1C, KDM2B, phospho-Rb expression in Z-138 and Granta-519 cell lines; EZH2-OE (-) lane is a vector control transfected lane, complete blot and exposures used in Suppl Fig. S7A and S7B. **G** Protein expression of CDKN1C, EZH2, KDM2B, and phospho-Rb upon CDKN1C knockdown in the presence of erdafitinib in Z-138 and Granta-519 cell lines; si-CDKN1C (-) lane is scrambled si-RNA transfected lane. **H** Heatmap for qPCR analysis in Z-138 cells shows rescue of E2F1 target genes previously downregulated by erdafitinib by now knocking-down CDKN1C in the presence of erdafitinib. **I** Co-immunoprecipitation of CDKN1C and E2F1 followed by western blot in Granta-519 and Z-138 cells. Model showing proposed hypothesis of CDKN1C mediated regulation of E2F1 target genes.

CDKN1C (p57^Kip2^) is a tumor suppressor gene and inhibits cyclin-CDK complex activity [46], thereby regulating the retinoblastoma RB-E2F pathway [49]. Since Rb phosphorylation and its subsequent dissociation from transcription factor E2F1 (Fig. 5A) is a significant determinant of cell-cycle progression from the G1-phase [50], we found Rb-phosphorylation was reduced upon FGFR1 loss in MCL cells compared to controls (Fig. 5B). Similar results were obtained by treating MCL cells with erdafitinib (Fig. 5C). As shown in Fig. 5D, transactivation of the representative bona fide E2F1 target genes (*CDK1, TK1, UHRF1, PCNA, TUBA1B* [51–54]), which are also part of the PSG in MCL, were down-regulated in shFGFR1 cells. Similar results were obtained by treating the MCL cells with erdafitinib (Fig. 5E), strengthening our observation that loss or inhibition of FGFR1 regulates E2F1-mediated transactivation. Further, ectopic expression of EZH2 in erdafitinib-treated MCL cells restored the expression of phosphorylation of RB, KDM2B, and downregulated CDKN1C protein levels (Fig. 5F). As expected, the loss of CDKN1C in erdafitinib-treated MCL cells restored the expression of RB and its phosphorylation (Fig. 5G) and upregulation in E2F1 target gene expression (Fig. 5H) with no change in levels of E2F1 (Fig. S6A). We further confirmed that CDKN1C immunoprecipitated E2F1 in MCL cells (Fig. 5I), suggesting that CDKN1C may regulate the expression of E2F1-mediated transactivation by direct binding and act as a repressor of E2F1 function.

### FGFR1 contributes to a feedback loop to regulate MYC stability, which regulates the EZH2-CDKN1C axis

**Fig. 6:**
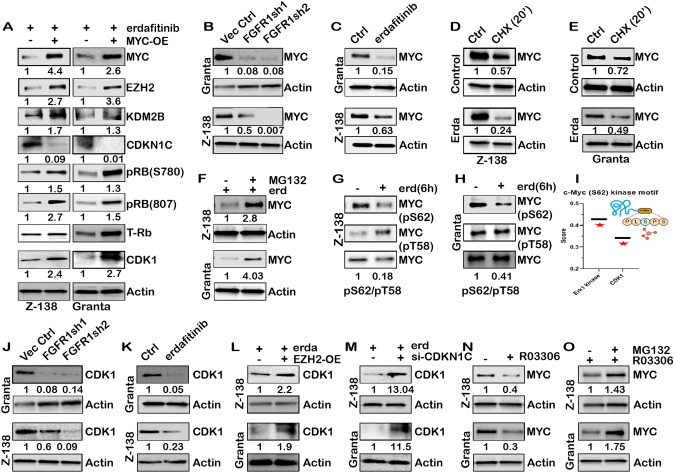
FGFR1 contributes to a feedback loop to regulate MYC stability, which regulates the EZH2-CDKN1C axis. **A** Z-138 and Granta-519 cells were transfected with MYC overexpression plasmid in the presence of erdafitinib treatment, and expression of EZH2, KDM2B, CDKN1C, phospho-Rb, CDK1 was evaluated. MYC-OE (-) lane is vector control transfected alone, complete blot and exposures used in Suppl Fig. S7A and S7B. **B** MYC protein expression upon FGFR1 knockdown and (**C**) erdafitinib treatment in Z-138 and Granta-519 cells, Actin in (**B**) is same as Fig. 5B; the same blot was used for MYC and Rb status in this case. **D** Z-138 and (**E**) Granta-519 cells were treated with 100ug/ml cycloheximide (CHX) for 20 min, with or without pre-treatment with erdafitinib. MYC protein expression was subsequently evaluated. **F** Z-138 and Granta-519 cells were treated with erdafitinib and supplied with 10uM proteasomal inhibitor MG-132. MYC expression was then determined. erdafitinib alone treatment lane for Granta-519 was split for representation; See complete blot in Suppl Fig. S7C; **G** Blots showing phospho-MYC Ser62 and Thr58 expression and ratio upon six hr. treatment with erdafitinib in Z-138 and (**H**) Granta-519 cells. **I** ScanSite 4.0 motif scan of Ser62 kinase motif of the human MYC protein revealed Erk1 kinase and CDK1 as top hits with the highest score. **J** CDK1 protein expression in FGFR1 knockdown and (**K**) erdafitinib treated cells of Z-138 and Granta-519. Actin in (**J**) is the same as in Fig. 3G, as the same blots were used for re-probing EZH2 and CDK1 in these cases. **L** Blots showing CDK1 expression upon EZH2 rescue in the presence of erdafitinib in Z-138 and Granta-519; complete blot and exposures used in Suppl Fig. S7A and S7B. **M** Blots showing CDK1 expression upon CDKN1C knockdown in the presence of erdafitinib in Z-138 and Granta-519. Note: Actin in (**L**) and (**M**) is the same as Fig. 5F and G, respectively, as the same blot was used for probing CDK1 in this case. **N** MYC expression upon treatment of Z-138 and Granta-519 cells with CDK1 inhibitor RO-3306. **O** Z-138 and Granta-519 cells were treated with RO-3306 and supplied with 10uM proteasomal inhibitor MG-132. MYC protein expression was then determined. RO-3306 alone treatment lane for Granta-519 was split for representation; See complete blot in Suppl Fig. S7D.

**Fig. 7:**
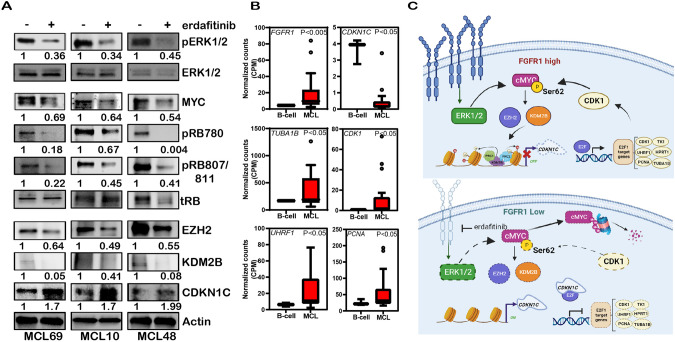
Orthogonal validation of FGFR1 mediated regulation of MYC-EZH2-CDKN1C axis- in MCL. **A** Western blots showing expression of p-Erk1/2, MYC, p-Rb, EZH2, KDM2B, and CDKN1C upon erdafitinib treatment of 3 MCL Patient-derived xenografts ex-vivo. **B** Tukey plots showing FGFR1, CDKN1C, TUBA1B, CDK1, UHRF1, and PCNA expression in 20 MCL patients compared to healthy donor B-cells. **C** Model showing the role of FGFR1 regulating feedback loop in MCL.

MYC regulates the expression of EZH2 [55] and KDM2B [56]; hence, we asked if EZH2 and KDM2B expression in erdafitinib-treated MCL is MYC dependent. As shown in Fig. 6A, MYC introduction in erdafitinib-treated MCL cells causes higher levels of expression of KDM2B and EZH2 and subsequent loss of CDKN1C along with reactivation of CDK1 (RB/E2F1 target), suggesting that FGFR1 mediated regulation of KDM2B/EZH2/CDKN1C is MYC dependent. Interestingly, the expression of MYC is significantly lower in either shRNA FGFR1 or erdafitinib-treated MCL cells compared to control (Fig. 6B-C, Fig. S6D), but the expression of *MYC* was unaltered (Fig. S6B-C). To investigate if the loss of MYC expression was due to a decreased stability of MYC protein with FGFR1 inhibition, we treated MCL cells with cycloheximide and determined that MYC degraded faster in the presence of erdafitinib (Fig. 6D-E). Further, treatment with the proteasomal inhibitor MG132 rescued the expression of MYC in erdafitinib-treated MCL cells (Fig. 6F). The phosphorylation on Serine 62 and Threonine 58 residues largely governs MYC stability, and a higher Ser62/Thr58 ratio indicates greater MYC stability [57–59]. We found that the MYC p-Ser(62)/p-Thr(58) ratio is reduced within 6 h of the addition of erdafitinib (Fig. 6G-H). Further, motif scan analysis on MYC found an S/TPx(x)R/K consensus motif on Serine 62 with high-affinity binding scores to two kinases, Erk1 and CDK1 (Fig. 6I). Both p-Erk1/2 and CDK1 levels are reduced upon FGFR1 knockdown and erdafitinib treatment (Fig. 6J, K; Fig. S6E-F). Activated Erk has been established to increase MYC stabilization through Ser62 phosphorylation [57, 60]. When MCL cells were treated with U0126, a MEK1/2 inhibitor that inhibits Erk’s activation, and we found a decrease in MYC expression, consistent with previous observations (Fig. S6G). CDK1 is downregulated in FGFR1 inhibition/loss in MCL cells (Fig. 6J-K) and restored by EZH2 overexpression (Fig. 6L) or CDKN1C downregulation (Fig. 6M) in erdafitinib treated MCL cells. Further, inhibiting the activity of CDK1 using RO-3306, a selective CDK1 inhibitor, we observed subsequent reduction in MYC levels (Fig. 6N) and the MYC p-Ser62/p-Thr58 ratio (Fig. S6H), bolstering the finding that CDK1 regulates MYC stability. Next, the MYC levels were restored by proteasomal inhibition using MG-132 in RO-3306 treated cells (Fig. 6O) or U0126 treated MCL cells (Fig. S6I). The orthogonal validation of our axis was performed in an ex-vivo culture of isolated PDX splenocytes treated with erdafitinib, confirming the loss of MYC, KDM2B, EZH2, CDK1, pERK1/2, pRB, and upregulation of CDKN1C, strengthening our in-vitro results (Fig. 7A; Fig. S6J). Further analysis of the RNA Seq data from healthy donors and MCL primary patients showed a lower expression of *CDKN1C* and higher expression of *FGFR1* and E2F target genes (*CDK1, PCNA, UHRF1, and TUBA1B*) in MCL primary patients (Fig. 7B).

## Discussion

FGFR1 activation is a known driver in various malignant diseases such as breast, prostate, myeloproliferative neoplasms, AML, ALL, CML, glioblastoma, and lung cancer [61–65]. Therapeutic targeting of FGFRs in cancers is an active area of research. TKI targeting the kinase domain of FGFRs is currently being tested in clinical trials [66, 67]. In particular, the FGFR-selective TKIs, including JNJ42756493 (erdafitinib), BGJ398 (NVP-BGJ398), and AZD4547, are currently under clinical evaluation on different oncotypes [66–68]. Our data here support that the selective inhibitor erdafitinib has pre-clinical activity in-vivo in murine models of MCL and presents an opportunity to characterize the potential of TKI targeting in MCL patients. FGF2-mediated activation of FGFR1 promotes resistance to EGFR inhibitors in lung cancer [69], FLT3 inhibitors in AML [70], and KIT inhibitors in gastrointestinal stromal tumors [71]. Noteworthy, FGFR1 can be activated via various ligands (FGF1-6, FGF-8, 19,21, and 23) [72], and previous studies of leukemia and lymphoma cells show higher expression of FGF2 ligands [73]. Recently, a study [74] shows higher levels of FGFR1 in MCL patients who did not respond to Ibrutinib. Our data show that FGFR1 expression correlates with poor prognosis in MCL patients who received CHOP/R-based therapy.

Similarly, the Ki-67 score alone stratified patient outcome, and in Ki67^+^ cases, FGFR1 continued to show significant adverse effects on overall survival. The data suggest that the prognostic effect of FGFR1 is independent of Ki67, but a future comparative analysis of FGFR1 and Ki-67 index using a more extensive but similarly treated patient cohort is warranted. Additionally, in a larger cohort, the adverse prognostic effects of *FGFR1* were observed in cases with high proliferation gene signature (PSG) compared to *PSG*^*low*,^ suggesting that FGFR1 has independent adverse prognostic effects on overall survival in MCL patients. Recent findings of FIGHT-101 [75] using pemigatinib shows that the most frequent alterations co-occurring with FGFR amplifications were TP53 alterations, however genomic analysis of MCL patients (n = 162) did not show any FGFR1 activating mutations. In addition, our data also support that FGFR1 expression can be regulated by stromal cells, thus postulating that both cell intrinsic and extrinsic factors contribute to FGFR1 expression and its activity in MCL.

Coordinated efforts of the polycomb repressor complexes PRC1 and PRC2 are known to maintain epigenetic repression at gene target loci. Canonical PRC1 contains four core subunits: PCGF4, PCGF2, PHC, CBX, and RING1A or RING1B, with E3 ubiquitin ligase activity on histone H2A at lysine-119, thereby inducing the compaction of chromatin [76]. In addition non-canonical PRC1-variants (PRC1.1,PRC1.3,PRC1.5, and PRC1.6) containing a distinct PCGF subunit have been identified [77]. Interestingly, we did not observe any change in protein expression of other PRC1 members other than the PRC1.1 member KDM2B in erdafitinib-treated MCL cells, raising the possibility of KDM2B regulation by FGFR1 in MCL. Ectopic expression of KDM2B in FGFR1 knockdown MCL cells restored EZH2 expression, confirming KDM2B to be downstream of FGFR1, consistent with previous reports [78].

Conversely, the loss of FGFR1 released suppression on CDKN1C, this epigenetic repression of CDKN1C in MCL is consistent with a previous report [79]. Our results indicate an intricate regulation of KDM2B and EZH2 to regulate the repression of CDKN1C in an FGFR1-dependent manner. Interestingly loss of CDKN1C in erdafitinib-treated cells also up-regulated the expression of EZH2, confirming the regulation of Rb-E2F1 transactivation by CDKN1C, as EZH2 expression is regulated by E2F1 [80]. On the contrary, CDKN1C loss in erdafitinib treated cells failed to up-regulate the expression of KDM2B, suggesting that its regulation may be Rb-E2F independent, possibly through EZH2, as ectopic expression of EZH2 restored the expression of KDM2B in erdafitnib treated MCL cells (Fig. 5F). However, a deeper investigation is warranted to delineate whether recruitment of KDM2B and EZH2 to target loci follows a ‘hierarchical’ model or ‘alternative model’ of chromatin assembly in MCL [76, 81–84].

Our observation is consistent with a previous report that shows G1 arrest upon inhibition of EZH2 through reactivation of p57(CDKN1C) [85]. Interestingly, upon FGFR1 inhibition, levels of total Rb protein were also reduced and subsequently increased upon rescue by EZH2 or loss of CDKN1C, suggesting that RB might be FGFR1 dependent. Our findings indicate that CDKN1C binding to E2F1 in MCL cells may be a repressor of E2F1-mediated transactivation. A recent report discusses transcriptional reprogramming via CDK9 in IR MCL [86], and another report shows that EZH1/2 inhibition overcomes ibrutinib resistance in MCL primarily by up-regulating CDKN1C [79]. The previous report shows CDKN1C binding to CDK7/9 inhibits transcription by preventing RNA Pol-II CTD phosphorylation [49]. Our data here show that FGFR1 signaling contributes to the suppression of CDKN1C via epigenetic regulation in MCL and may be targeted in ibrutinib-resistant patients, but this hypothesis requires further validation.

Overexpression of MYC has been associated with an aggressive blastoid MCL variant [87, 88], and MYC cooperates with Cyclin D1 to drive mouse lymphomagenesis [15, 89, 90]. Consistent with these reports, the introduction of MYC in erdafitinib-treated cells resulted in up-regulated EZH2 and KDM2B. Interestingly ectopic expression of EZH2 and loss of CDKN1C also increased levels of MYC (Fig. S6K), suggesting that epigenetic regulation in MCL may constitute a feedback loop. Further, we show MYC stability depends on phosphorylation at serine 62 [60], and either FGFR1 or CDK1 inhibition in MCL cells decreased S62 phosphorylation. In addition, our data indicate that the downstream mediator of FGFR1 signaling, pERK1, also promotes MYC stability, which eventually results in CDKN1C-mediated regulation of CDK1 and can, in turn, regulate the stability of MYC, resulting in the maintenance of a feedback loop. This possibly explains why CDKN1C downregulation in the presence of the FGFR1 inhibitor also increases MYC levels.

In summary, our data provide a model of cellular proliferation in MCL that is dependent upon FGFR1 levels and shows that FGFR1 loss up-regulates the expression of CDKN1C via MYC/KDM2B/EZH2 axis and regulates E2F target gene expression such as CDK1, which forms a feedback loop to maintain MYC levels and provide a proliferative advantage to MCL cells (Fig. 7C). Further, we show that FGFR1 is therapeutically targetable with erdafitinib to achieve lower tumor burdens in multiple in-vivo MCL models to improve overall survival. This report provides the first compelling pre-clinical rationale for targeting FGFR1 in MCL.

## Availability of data materials

Data sets used or generated in this study can be accessed through the GEO portal using the accession numbers mentioned in respective methods.

### Supplementary information

Supplementary methods and Figures

Supplementary Table 6

Supplementary Table 1

Supplementary Table 2

Supplementary Table 3

Supplementary Table 4

Supplementary Table 5

Supplementary Table 7

Supplementary Table 8

## Data Availability

Data sets used or generated in this study can be accessed through the GEO portal using the accession numbers mentioned in respective methods.
