# Supplementary material for: Exploiting the fibroblast growth factor receptor-1 vulnerability to therapeutically restrict the MYC-EZH2-CDKN1C axis-driven proliferation in Mantle cell lymphoma
Source: Leukemia. 2023 Aug 19;37(10):2094–106. doi: 10.1038/s41375-023-02006-8 (PMC10539170; doi:10.1038/s41375-023-02006-8)

**Exploiting the Fibroblast Growth Factor Receptor-1 vulnerability to therapeutically restrict the** **MYC-EZH2-CDKN1C axis-driven proliferation in Mantle Cell Lymphoma**

Anuvrat Sircar,^1,2*^ Satishkumar Singh,^1,2*^ Zijun Y. Xu-Monette,^3^ Krysta Mila Coyle,^4^ Laura K Hilton, ^5^ Evangelia Chavdoula,^2,6^ Parvathi Ranganathan,^1,2^ Neeraj Jain,^7,8^ Walter Hanel,^1,2^ Philip Tsichlis,^2,6^ Lapo Alinari,^1,2^ Blake R. Peterson,^2,9^ Jianguo Tao,^10^ Natarajan Muthusamy,^1,2^  Robert Baiocchi,^1,2^ Narendranath Epperla,^1,2^ Ken H. Young,^3,11^ Ryan Morin,^4,12^ and Lalit Sehgal^1,2,🖂^

**Supplementary Materials and Methods:**

**Cell culture:** MCL cell lines were cultured in RPMI media (ThermoFisher:11875) supplemented with 10% Fetal Bovine Serum (FBS) (ThermoFisher:10437028) and 1X Penicillin-Streptomycin (PS)(Gibco: 15140122). HS-5 cells were cultured in DMEM media (ThermoFisher: 10569044) supplemented with 10% FBS and 1XPS. For passaging HS-5 cells, media was removed, and cells were washed with 1X Phosphate buffered saline (PBS), followed by trypsinization using Trypsin-EDTA (0.25%), phenol red (ThermoFisher: 25200056), neutralization with fresh media, and subsequent plating. All parental cell lines were regularly tested for mycoplasma (Lonza) and authenticated by STR profiling. MCL cell lines (Z-138, Jeko-1, SP-53, SP49, Mino, and Granta-519) were maintained per the manufacturer’s protocol; the resistant cells were cultured as described previously^1,2^, and Fc-muMCL1 was a gift from Dr. Christopher Smiths.

**Cell cycle analysis and apoptosis assay:** Cells were treated with erdafitinib, tazemetostat, or valemetostat for 16h and subsequently washed with PBS, fixed in ice-cold 70% ethanol, and stained with FxCycle PI/RNase Staining Solution (Catalog no. F10797) for 20 min. Stained cells in duplicate replicates were then acquired and analyzed in the BD LSRFortessa Cell Analyzer Flow cytometer for the cell cycle. The Synchronous cell with transient knockdown was acquired and analyzed for cell cycle using two different cell cycle models (Watson and Dean-Jett), and values obtained were plotted in GraphPad. For measuring apoptosis, Annexin V-CF Blue 7-AAD Apoptosis Staining / Detection Kit (Abcam, ab214663) was used per the manufacturer’s instructions. CF Blue fluorescence was measured in the FL1 (Ex/Em max = 408/450nm) channel and 7-AAD fluorescence in the FL3 (Ex/Em max = 543/647nm) channel. In addition, the Early Tox live/dead assay kit (Molecular Devices) was used per the manufacturer’s protocol.

**Cell-proliferation assay:** 10^4^ cells were seeded in each well of a 96-well plate. The WST -1 reagent was added every 24h, and normalized absorbance was recorded (OD 450nm/690nm). After the 72h time-point, normalized absorbance was plotted with respect to time on the XY axis (non-linear regression) or the area under the curve (AUC) as calculated using GraphPad Prism 9.

**Immunoblotting:** Cells were harvested and lysed in ice-cold 1X SDS cell lysis buffer supplemented with Halt Protease and Phosphatase Inhibijetor Cocktail from ThermoFisher. Total protein was quantitated using Pierce BCA Protein Assay Kit (Fisher Scientific, Cat. 23225). Then, an equal amount of protein was loaded, resolved through sodium dodecyl sulfate-polyacrylamide gel electrophoresis, transferred onto PVDF membrane (BioRad), incubated with different primary and corresponding HRP-conjugated secondary antibodies (List of antibodies in supplemental methods), and analyzed using either WesternBright ECL HRP Substrate (Advansta, K-12045) or SuperSignal West Femto Maximum Sensitivity Substrate (Fisher Scientific, Cat. 34095) in ChemiDoc Imaging system (Biorad, #12003153). Blots were stripped using Restore Western blot stripping buffer as needed (Thermo, #21059), re-blocked with 5%BSA, and probed for desired antibodies. Details on the antibodies used are listed below.

| **Protein** | **Company** | **Catalog** |
| --- | --- | --- |
| FGFR1 | Abcam | ab76464 |
| EZH2 | CST | 5246 |
| MYC | CST | 18583 |
| CDKN1C | CST | 2557 |
| p-Erk (T202/Y204) | CST | 4370 |
| Total Erk | CST | 4696 |
| KDM2B | Millipore | 09-864 |
| p-Rb (S807/811) | CST | 8516 |
| p-Rb (S780) | CST | 9307 |
| Total Rb | Santa Cruz | sc-7905 |
| p-MYC Ser62 | CST | 13748 |
| p-MYC Thr58 | CST | 46650 |
| CDK1 | CST | 9116 |
| E2F1 | CST | 3742 |
| Actin | Sigma | A3854 |
| H3 | CST | 4499 |
| SUZ12 | CST | 3737S |
| PCGF4 | CST | 6964 |
| H3K27me3 | CST | 9733S |
| pMEK1/2 | CST | 9154S |
| MEK1/2 | CST | 8727S |
| pAKTS473 | CST | 9271S |
| AKT | CST | 9272S |
| p-mTOR S2448 | CST | 5536P |
| mTOR | CST | 2983P |
| GAPDH | CST | 5174T |
| EZH1 | CST | 42088S |
| Cleaved PARP | Abcam | ab32064 |
| RING1A | CST | 13069 |
| RING1B | CST | 5694S |
| PCGF1 | ThermoFisher | PA5-49390 |
| Bmi1 | CST | 6964S |

**Immunoprecipitation:** For immunoprecipitation of protein-protein complexes, cells were washed in cold PBS, and harvested cells were lysed in ice-cold 1X non-denaturing cell lysis buffer (CST, 9803) supplemented with PMSF followed by sonication. Protein concentration was quantified, and cell lysate was pre-cleared. 200ug of cell lysate was incubated with EZH2 (D2C9) Rabbit monoclonal antibody (CST#5246) at a ratio of 1:200 and incubated overnight at 4°C with rotation. Prewashed protein A magnetic beads (CST,73778) were mixed with the lysate-antibody (immunocomplex) solution and incubated at RT with rotation. Beads were pelleted with a magnetic separation rack and washed with ice-cold cell lysis buffer five times. 30µl of 3X SDS sample buffer was added to beads and mixed well. The sample was heated at 95°C, beads separated, and the supernatant was transferred to a fresh tube. The sample was then analyzed by western blotting as described above.

**Quantitative Real-time PCR:** Total RNA was isolated using Qiagen RNeasy Plus Mini (74104) or Micro (74004) Kit per the manufacturer’s instructions. cDNA was synthesized from extracted RNA using SuperScript III First-Strand Synthesis System (Fisher Scientific, Cat. 18080051). Then, quantitative PCR was performed using the CFX96 real-time PCR system (BioRad) utilizing PowerUp SYBR Green Master Mix (Applied Biosystems, A25741). First, normalization was carried out with respect to β-actin in duplicate, followed by the calculation of average relative quantity (RQ). Taqman Probes were used, FGFR1 Taqman probe 4331182(Hs00241111_m1), FGFR2 Taqman probe4331182 (Hs01552918_m1), FGFR3 Taqman probe 4331182 (Hs00179829_m1) and FGFR4 Taqman probe4331182 (Hs01106910_g1.)

**Chromatin immunoprecipitation or CUT & RUN:** ChIP cells were cross-linked by incubation with 1% formaldehyde (Sigma Aldrich) at 37°C for 15 minutes. The reaction was stopped by adding glycine (0.125 M final concentration; Sigma Aldrich) and processed for chromatin isolation using specific antibodies (details of antibodies in supplemental methods) followed by RT PCR described previously.^3^ Cleavage under targets and release using nuclease (CUT&RUN) was performed per the protocol described^4^ for KDM2B, EZH2, CUT&RUN PAG-MNase, and Spike-In DNA #40366 (Cell Signaling) was used as cleavage and a spike in control. The Seq was performed on hi-Seq2500. The Seq data were analyzed using the Basepair CUT&RUN pipeline to identify the common peaks for KDM2b and EZH2.

**Gene Expression Studies and Analysis:** Z138 Vec ctrl and shFGFR1 cells were used in triplicate for the gene expression studies. Briefly, the total RNA from each sample was quantified using the NanoDrop ND-1000 spectrophotometer. RNA integrity was assessed by standard denaturing agarose gel electrophoresis. For microarray analyses, the total RNA from each sample was amplified and transcribed into fluorescent complementary RNA using the manufacturer’s Quick Amp Gene Expression Labeling Protocol, Version 5.7.9 (Agilent Technologies, Santa Clara, CA, USA). The labeled complementary RNAs were hybridized onto a whole human genome oligo microarray (4 3 44K; Agilent Technologies). After washing the slides, the arrays were scanned using the Agilent microarray scanner G2505C. Agilent’s Feature Extraction software (version 11.0.1.1) was used to analyze array images. Quantile normalization and subsequent data processing were performed using the GeneSpring GX v12.1 software (Agilent Technologies). Differentially expressed genes were identified through fold-change and volcano filtering. Agilent’s pathway and Gene Ontology analyses were applied to determine the roles these differentially expressed genes play in these biological pathways or Gene Ontology terms. Finally, a Venn diagram was generated to show samples’ distinguishable gene expression profiles. Granta-519 Vec ctrl or shFGFR1 were used for the RNA Sequencing studies; RNA Isolation was performed as described above, followed by QC using a bioanalyzer. The total stranded library was generated and run on NovaSeq paired-end 150bp. Sequencing files were uploaded to Basepair and were analyzed for downstream analysis. The RNA-Seq data were analyzed using Basepair software (<https://www.basepairtech.com>) with a pipeline that included the following steps. First, reads were aligned to the transcriptome derived from UCSC genome assembly (hg19) using STAR^5^ with default parameters. Second, read counts for each transcript were measured using featureCounts.^6^ Differentially expressed genes were determined using DESeq2^7^, and a cut-off of 0.05 on the adjusted p-value (corrected for multiple hypotheses testing) was used for creating lists. Third, GSEA was performed on normalized gene expression counts, using gene permutations for calculating the p-value. Finally, we deposited the corresponding raw data into the Gene Expression Omnibus data repository under accession numbers GSE138127 and GSE223954.

**Dataset Mining and Analysis:** log2 mRNA expression of FGFR1-4 was analyzed using R2 genomics with validated probe sets 211535_s_at for FGFR1^8-11^, 208228_s_at for FGFR2^10,12^ 204379_s_at for FGFR3^13^ 204579_at for FGFR4^14^ described previously in E-MEXP-2360, E-MTAB-1771, GSE93291. FGFR1 expression (averaged for Uid: 32297,27758,27747,27445 and 17271) and Proliferation gene signature average and associated survival information were downloaded from LLMP, RNA expression cut-off was determined using the log-rank test and survival graphs were plotted using GraphPad. In addition, bulk transcriptome analyses were analyzed by Basepair software (https://www.basepairtech.com/) differential expression pipeline to identify critical upregulated genes (RNA-seq datasets GSE55405, GSE46846, GSE99501). For comparative analysis between MCL and healthy donors, GSE46846 and GSE70926 were queried in GREIN to obtain the Normalized count (CPM). RNA sequencing data for the MCL treated with CHOP/R (≤6 cycles n=30) in the Morin cohort was obtained from previously published work^15^ Briefly, gene expression levels were quantified from fastq with Salmon and normalized with DESeq2 and variance stabilizing transformation. Expression matrices underwent batch correction to remove artifacts related to the source material: frozen whole tissue, immunotubes, and FFPE. Survival information was extracted for patients with CHOP/R treatment (<=6 cycles). For mutational analysis, 111 genomes and 51 exomes from previously published studies were aligned to the hg38 reference genome^15,16^. Mutations were detected as previously described^17^. Briefly, mutations were called using the SLMS-3 pipeline that amalgamates Strelka2^18^, SAGE, Lofreq^19^, and mutect2^20^ to report somatic variants supported by at least three algorithms. The consistent cut-off to call the variants was >=4 reads and >=0.1 VAF. The detected variants were further annotated using the command line vcf2maf (version 1.6.18) and Variant Effect Predictor (cache version 86).

**CUT & RUN Data Processing:** The raw fastq data was analyzed using Basepair software with a pipeline that included the following steps: Raw reads were trimmed using fastp^21^ to remove low-quality bases (quality < 20) and adapter sequences. Reads less than 15bp were discarded, and the remaining reads were aligned with Bowtie2^22^ to hg19 genome assembly in a very-sensitive mode. Alignments were sorted and indexed using samtools,^23^ deduplicated using sambamba,^24^ and mitochondrial reads were removed. Genome-wide read coverage was computed using bedtools^25^, and the insert-size distribution was calculated with the Picard toolkit (<http://broadinstitute.github.io/picard>). Using deeptools,^26^ alignments were performed to generate RPKM-normalized heatmaps and average profiles, and data were visualized using the IGV browser^27^, and peaks were identified with MACS2^28^ using a p-value threshold of 1e-5, and functional enrichment analyses of peaks were performed with Homer.^29^ Peaks overlapping blacklisted regions were discarded, and remaining filtered peaks were annotated based on their proximity to gene features, where peaks between −2500 and 2500 bp of a transcription start site were marked as “Promoter,” overlapping “Exon” or “Intron” and the rest were marked as “Intergenic.” For intergenic peaks, a gene was considered a target if it was within 1 Mb of the peak. The analyzed data is presented as a supplementary table. Raw data from the ATAC Seq on Z138 GSM3473048 was processed using Basepair and overlapped with the genome browser to confirm the accessibility of the genomic region identified by CutnRun.

**Statistical analysis:** Unless otherwise indicated, experimental data are reported as means ± SEM. Group differences were calculated using (GraphPad Prism, La Jolla, CA). All power calculations assume a two-sided test and the Bonferroni method for controlling type I error at 0.05. The calculated sample sizes provide at least 80% power to detect the specified differences (a widely accepted biological difference unless otherwise stated). For the animal studies with similar genetic backgrounds (NSG mice), n≥5 replicates per group/cell line was used to provide 80% power to detect a significant change for up to five outcome measurements at CV=20% (α=0.05/5). Statistical analyses such as one- or two-sided, ANOVA, coefficient of Variation, Covariates, assumptions of corrections, and distributions were calculated by GraphPad and described where applicable in the figure. Similarly, for the studies with patient samples such as viability or protein expression, n≥6 samples were used, as patient samples generally have larger variation. A p-value less than 0.05 was considered statistically significant. Statistical analysis used for each experiment is mentioned in the figure legends. For the survival studies, a Log-rank test was used.

**Tissue microarray and immunohistochemistry:** For IHC on human samples, hematoxylin-eosin-stained slides from each of the 31 cases of MCL were reviewed, and TMA was constructed from tumor cell-rich areas. IHC studies for various markers using a streptavidin-biotin complex technique were performed on 4-µm TMA sections. The markers assessed included: FGFR1, Ki67, and CyclinD1. A cut-off value for each marker was established from the analysis of receiver-operating characteristic curves to achieve maximum specificity and sensitivity. According to the manufacturer’s instructions, immunohistochemistry for FGFR1 was performed on formalin-fixed paraffin-embedded tissues using an anti-FGFR1 antibody (Abcam, ab76464). In addition, the Immunohistochemistry analysis was performed on the archival MCL tissues by KHY and XYZM, and the scores were plotted and correlated.

**Xenograft study:** Animal studies were completed under protocols for animal welfare approved by the Institutional Animal Care and Use Committee, The Ohio State University, Columbus, OH, USA). Briefly, 6-week-old male or female NSG mice (Target validation core, OSU) were subcutaneously injected with MCL cells mixed with Matrigel matrix (Corning) in a 1:1 ratio on the flanks. Erdafitinib powder was dissolved in a 10% HP-beta-cyclodextrin solution. Mice were randomized 5-7 days after transplantation, and erdafitinib solution (12.5mg/kg) was administered by oral gavage to mice once per day. Tumor volume was periodically measured using calipers, and tumor volume was calculated as described previously.^30^ Mice were sacrificed when early removal criteria (ERC) were met. Mice showing no engraftment were excluded from the study and were not considered in statistical analyses. In co-transplant experiments of Jeko-1 and HS5 cells, 2 million Jeko-1 cells were mixed with 0.75 million HS5 cells and subcutaneously transplanted with Matrigel in NSG mice. In addition, 0.75 million HS5 cells transplanted with Matrigel alone acted as a control. Investigators were not blinded for animal studies.

**Plasmids and transfections:** FGFR1 shRNA constructs cloned in pGIPZ and pLKO.1 vector was used for generating FGFR1 knockdown (sequences used are available in supplementary methods). shRNA EZH2, shKDM2B, WT-KDM2B, WT-EZH2, and WT-MYC constructs were gifts from Dr. Philip Tsichlis. The transfection of the plasmids was performed using the Calcium chloride-BBS method, and the transduction was performed as described previously.^31^ Below is the List of Primers, shRNA Sequences, and used reagents.

| shRNA/siRNA constructs |  |  |
| --- | --- | --- |
| FGFR1 | Dharmacon | RHS4430-200230856, V3LHS_634644 |
|  |  | RHS4430-200224857, V3LHS_634642 |
| FGFR1 | Millipore sigma | TRCN0000121182 |
|  |  | TRCN0000121185 |
| CDKN1C | Horizon discovery | L-003244-00-0005 |
| **Gene** | **Forward** | **Reverse** |
| EZH2 | 5’ CTGATTTTACACGCTTCCGC 3’ | 5’ GGAACAACGCGAGTCGG 3’ |
| MYC | 5’ CACCGAGTCGTAGTCGAGGT 3’ | 5' TTTCGGGTAGTGGAAAACCA 3' |
| CDKN1C | 5’ AGATCAGCGCCTGAGAAGTCGT 3’ | 5’ TCGGGGCTCTTTGGGCTCTAAA 3’ |
| Actin | 5’ CATGTACGTTGCTATCCAGGC 3’ | 5' CTCCTTAATGTCACGCACGAT 3' |
| CDK1 | 5' GGATGTGCTTATGCAGGATTCC 3' | 5' CATGTACTGACCAGGAGGGATAG 3' |
| HPRT1 | 5’ CCTGGCGTCGTGATTAGTGAT 3’ | 5’ AGACGTTCAGTCCTGTCCATAA 3’ |
| PCNA | 5' GCGTGAACCTCACCAGTATGT 3' | 5' TCTTCGGCCCTTAGTGTAATGAT 3' |
| TK1 | 5’ GGGCAGATCCAGGTGATTCTC 3’ | 5' TGTAGCGAGTGTCTTTGGCATA 3' |
| TUBA1B | 5’ ACCTTAACCGCCTTATTAGCCA 3’ | 5’ ACATTCAGGGCTCCATCAAATC 3’ |
| UHRF1 | 5' AGGTCAATGAGTACGTCGATGC 3' | 5’ TTCTCCGGGTAGTCGTCGT 3’ |
| CDKN1C ChIP F1R1 | 5' TGGCTTTTGGTTCCACCATCA 3' | 5' AGTGGATGAGACAGGGCGTT 3' |
| CDKN1C ChIP F3R3 | 5’ TGGGTTAACGCCCTGTCTC 3’ | 5’ CTTTGAGTCCCAAGACACCCC 3’ |
| **Inhibitors Used** |  |  |
| Ibrutinib | Medchemexpress | HY-10997 |
| Valemetostat | Medchemexpress | HY-109108 |
| Tazemetostat | Selleckchem | S7128 |
| RO-3306 | Selleckchem | S7747 |
| PD173074 | Medchemexpress | HY-10321 |
| Erdafitinib | Janssen pharmaceuticals |  |
| MEKi (U0126) | CST | 9903S |

**Supplementary Tables**

Supplementary Table 1: Differential expression in MCL determined by bulk RNA sequencing analysis.

Supplementary Table 2: List of genes in the Venn Diagram.

Supplementary Table 3: Characteristics of MCL Patients, treatment, and Survival information.

Supplementary Table 4: Venn Diagram for the Differential expressed gene in shFGFR1 Knockdown MCL cells.

Supplementary Table 5: KEGG analysis for the Differential expressed gene in shFGFR1 Knockdown MCL cells.

Supplementary Table 6: ARCHS4 analysis on the Differential expressed genes.

Supplementary Table 7: Fold enrichment for EZH2 and KDM2B peaks in MCL.

Supplementary Table 8: Enricher Analysis of the common peaks in MCL.

**Supplementary Figure Legends:**

**Figure S1:** (A) WST-1 assay to determine the fold change in absorbance (a measure of cell viability) after 72h when Jeko-1, SP-53, MCL patient 48, MCL patient 69, or Fc-muMCL1 cells were cultured with or without HS-5 conditioned media. (B) The area under the curve (AUC) calculated after ibrutinib, acalabrutinib, or doxorubicin treatment to generate dose-dependent cell viability plot by absorbance evaluation after WST-1 addition in Jeko-1 and SP-53 cells grown with or without HS-5 conditioned media. (C) Live/dead cell ratio calculated after Calcein AM-EthD-III treatment of MCL patient 48 and patient 69 cells grown 24h in culture, with or without HS-5CM. *Unpaired T-test* (D) Venn diagram of the upregulated gene from RNA seq analysis (p<0.01, FC>1.6). (E) Log2 expression of four identified genes from panel (D) in MCL Patients (n=122) and COV was calculated with the slightest variation in FGFR1. (F) Western blots show that expression of FGFR1 protein in MCL cell lines SP-53 and Jeko-1 was increased when cultured under HS-5 stromal conditioned media (HS5-CM). FGFR1 protein expression in 2 patient samples with relatively lower FGFR1 expression is further elevated when cultured under HS5-CM. The MCL cell lines Jeko-1R and SP-49R with acquired ibrutinib resistance had a higher FGFR1 expression than ibrutinib-sensitive cells.

**Figure S2:** (A) FGFR1^-^ (<10% cutoff) patient samples had a lower Ki-67 expression (IHC%), while FGFR1^high^(≥10% cutoff) patient samples had a higher Ki-67 expression, indicative of more significant proliferation. *Unpaired T-test*. (B) Kaplan Meier plot for overall survival of patients segregated based on FGFR1-Ki67 protein expression in the Young cohort. *Log-rank mantel cox test* (1 vs. 3, p=0.128; 3 vs. 4, p=0.026; 1 vs. 4, p=0.0001) (C) Kaplan Meier plot for overall survival of patients segregated based on *MKI67^low/high^* expression (p=0.03) and (D) *FGFR1-MKI67* expression in CHOP/R treated patients, Morin cohort. (1 vs 2, p=0.07; 1 vs 3 p=0.025; 1 vs 4, p=0.002). (E) Waterfall plot showing a summary of *TP53* and *FGFR1* mutations in 162 MCL patients from previously published studies (*details in suppl. methods)*. (F) Kaplan Meier plot for overall survival of patients segregated based on *Proliferation gene signature (PSG^low/high^)* expression (p=0.0001) and (G) *FGFR1-PSG* expression in Rosenwald cohort. (1 vs 3, p=0.1; 3 vs 4, p=0.02; 1 vs 4, p=0.0001). (H) FGFR1 protein expression was reduced in shRNA-mediated FGFR1 knockdown in MCL cell lines Z-138, Jeko-1-R, Granta-519, and (I) SP-49R cells.

**Figure S3:** (A) FGFR2, (B) FGFR3, and (C) FGFR4 expression is not significantly altered among naïve or activated B-cells and 122 MCL patient samples. (D) FGFR1 expression is higher than other FGFR isoforms in 122 MCL patients and (E) MCL cell lines. (F) Annexin-7AAD staining analysis of MCL cells treated *in-vitro* for seven consecutive days with different concentrations of erdafitinib to show the percentage of live cells. *2-way ANOVA (a=0.05)*. (G) The area under the curve (AUC) calculated by WST-1 treatment of cells every 24h for 72h and plot of measured absorbance (indicative of cell viability) over time in Jeko-1 and (H) SP-53 cells grown either in HS-5CM or HS5CM supplemented with erdafitinib (5uM). *Ordinary one-way ANOVA*. (I) Tumor volume was measured at the ERC of the first mouse when either Jeko-1 or Jeko-1R cells were injected subcutaneously in NOD-SCID mice. (J) The Kaplan-Meier graph shows the mice survival described in panel (I).

**Figure S4:** (A) qPCR analysis shows a reduction in EZH2 mRNA expression upon FGFR1 knockdown in Z-138 and Granta-519 cells. *Ordinary one-way ANOVA*. (B) Percentage of G1 population in Z-138 and Granta-519 cells upon treatment with the EZH2 inhibitor tazemetostat (Taz) or the EZH1/2 inhibitor valemetostat (val), determined by cell cycle analysis. *2-way ANOVA (a=0.05).* (C) Western blots to show expression of other PRC2 and PRC1 complex members upon treatment with erdafitinib and PD-173074 in Z-138 and (D) Granta-519 cells. Note: Actin for erdafitinib treatment is the same as Fig5C, as the same blots were used for probing Rb status and proteins mentioned above in this case. (E) Western blots show decreased KDM2B protein expression upon FGFR1 knockdown in Z-138, Jeko-1R, and SP-49R cells. Note: Actin for Z-138 and SP-49R is the same as Fig3G, as the same blots were used for probing KDM2B and EZH2 in this case. (F) Western blots to show restoration of KDM2B and EZH2 upon KDM2B overexpression in Z-138 shFGFR1 cells.

**Figure S5:** (A) Depiction of KDM2B and EZH2 binding peaks obtained in Z138 cells. (B) ChIP-qPCR analysis for H3K27 tri-methylation at CDKN1C promoter regions in Granta-519, Jeko-1R, and SP49-R cell lines. The graph was plotted to illustrate enrichment in H3K27me3 antibody binding at depicted promoter regions compared to IgG isotype control antibody binding, and statistical analysis was performed using an *Unpaired T-test*. (C) Western blots show increased CDKN1C expression upon EZH2 knockdown in Jeko-1R cells. CDKN1C expression is increased upon KDM2B knockdown in Z-138 cells. (D) Western blots to show phospho-Rb expression in Jeko-1R and SP-49R cells upon erdafitinib treatment. Note: Actin for Jeko-1R is the same as Fig3H; the same blot was used to probe EZH2 and Rb status in this case.

**Figure S6:** (A) E2F1 expression in Granta-519 and Z-138 cells upon FGFR1 knockdown and erdafitinib treatment. (B) qPCR analysis shows *MYC* mRNA levels unchanged upon FGFR1 knockdown in Z-138 cells. (C) qPCR analysis shows *MYC* mRNA levels unchanged upon erdafitinib treatment in Z-138 and Granta-519 cells. (D) Protein expression of MYC upon FGFR1 knockdown in SP-49R cells. Note: Actin for SP-49R is the same as Fig S2, as the same blot was used for probing FGFR1 and MYC in this case. (E) p-Erk1/2 protein expression upon FGFR1 knockdown and (F) erdafitinib treatment in Z-138 and Granta-519 cells. Note: Actin for Granta-519 in panel (F) is the same as in Fig3H, as the same blot was used to probe p-Erk status and EZH2 in this case. (G) MYC expression upon treatment of Z-138 and Granta-519 cells with MEK inhibitor U0126. (H) p-MYC (Ser62) and p-MYC (Thr58) ratio in Z-138 and Granta-519 cells treated with CDK1 inhibitor RO-3306 for 6h. (I) Western blot to show rescue of MYC expression by proteasomal inhibitor MG-132 in U0126 treated Z-138 and Granta-519 cells. Note: U0126 alone treatment lane for Granta-519 is the same as in Fig S6G. See complete details in Suppl Fig S7E. (J) CDK1 protein expression upon erdafitinib treatment in two MCL PDX samples. Note: Continuation from Fig7A (K) MYC protein expression upon EZH2 overexpression or CDKN1C knockdown in erdafitinib-treated MCL cells. Note: Continuation from Fig5F and Fig5G. See complete blot and exposures used for MYC and EZH2 overexpression in Suppl. Fig S7A and S7B.

**Figure S7:** Blots as referred to: (A),(B) in Fig5F, Fig6A, Fig6L, and FigS6K, showing detailed loading order for MYC-OE, EZH2-OE, and vec control (VC-same for both MYC-OE and EZH2-OE since same expression plasmid used for cloning MYC and EZH2 OE constructs) in the presence of erdafitinib. Blots referred to: (C) in Fig6C and Fig6F, (D) in Fig6N and Fig6O, and (E) in FigS6G and FigS6I, showing detailed loading order for Granta-519 cells.

**References**

1 Zhao, X. *et al.* Unification of de novo and acquired ibrutinib resistance in mantle cell lymphoma. *Nat Commun* **8**, 14920, doi:10.1038/ncomms14920 (2017).

2 Zhao, X. *et al.* Transcriptional programming drives Ibrutinib-resistance evolution in mantle cell lymphoma. *Cell Rep* **34**, 108870, doi:10.1016/j.celrep.2021.108870 (2021).

3 Sehgal, L. *et al.* FAS-antisense 1 lncRNA and production of soluble versus membrane Fas in B-cell lymphoma. *Leukemia* **28**, 2376-2387, doi:10.1038/leu.2014.126 (2014).

4 Skene, P. J., Henikoff, J. G. & Henikoff, S. Targeted in situ genome-wide profiling with high efficiency for low cell numbers. *Nat Protoc* **13**, 1006-1019, doi:10.1038/nprot.2018.015 (2018).

5 Dobin, A. *et al.* STAR: ultrafast universal RNA-seq aligner. *Bioinformatics* **29**, 15-21, doi:10.1093/bioinformatics/bts635 (2013).

6 Liao, Y., Smyth, G. K. & Shi, W. featureCounts: an efficient general purpose program for assigning sequence reads to genomic features. *Bioinformatics* **30**, 923-930, doi:10.1093/bioinformatics/btt656 (2014).

7 Love, M. I., Huber, W. & Anders, S. Moderated estimation of fold change and dispersion for RNA-seq data with DESeq2. *Genome Biol* **15**, 550, doi:10.1186/s13059-014-0550-8 (2014).

8 Verhaak, R. G. W. *et al.* Mutations in nucleophosmin (NPM1) in acute myeloid leukemia (AML): association with other gene abnormalities and previously established gene expression signatures and their favorable prognostic significance. *Blood* **106**, 3747-3754, doi:10.1182/blood-2005-05-2168 (2005).

9 Messina, M. *et al.* Protein kinase gene expression profiling and in vitro functional experiments identify novel potential therapeutic targets in adult acute lymphoblastic leukemia. *Cancer* **116**, 3426-3437, doi:<https://doi.org/10.1002/cncr.25113> (2010).

10 Wöhrle, S. *et al.* Fibroblast Growth Factor Receptors as Novel Therapeutic Targets in SNF5-Deleted Malignant Rhabdoid Tumors. *PLOS ONE* **8**, e77652, doi:10.1371/journal.pone.0077652 (2013).

11 Tobias, B. *et al.* Transcriptional upregulation of p21/WAF/Cip1 in myeloid leukemic blasts expressing AML1-ETO. *Haematologica* **93**, 1728-1733, doi:10.3324/haematol.13044 (2008).

12 Lottaz, C. *et al.* Transcriptional profiles of CD133+ and CD133- glioblastoma-derived cancer stem cell lines suggest different cells of origin. *Cancer research* **70 5**, 2030-2040 (2010).

13 Hanamura, I., Huang, Y., Zhan, F., Barlogie, B. & Shaughnessy, J. Prognostic value of Cyclin D2 mRNA expression in newly diagnosed multiple myeloma treated with high-dose chemotherapy and tandem autologous stem cell transplantations. *Leukemia* **20**, 1288-1290, doi:10.1038/sj.leu.2404253 (2006).

14 French, D. M. *et al.* Targeting FGFR4 Inhibits Hepatocellular Carcinoma in Preclinical Mouse Models. *PLOS ONE* **7**, e36713, doi:10.1371/journal.pone.0036713 (2012).

15 Pararajalingam, P. *et al.* Coding and noncoding drivers of mantle cell lymphoma identified through exome and genome sequencing. *Blood* **136**, 572-584, doi:10.1182/blood.2019002385 (2020).

16 Nadeu, F. *et al.* Genomic and epigenomic insights into the origin, pathogenesis, and clinical behavior of mantle cell lymphoma subtypes. *Blood* **136**, 1419-1432, doi:10.1182/blood.2020005289 (2020).

17 Thomas, N. *et al.* Genetic subgroups inform on pathobiology in adult and pediatric Burkitt lymphoma. *Blood* **141**, 904-916, doi:10.1182/blood.2022016534 (2023).

18 Kim, S. *et al.* Strelka2: fast and accurate calling of germline and somatic variants. *Nat Methods* **15**, 591-594, doi:10.1038/s41592-018-0051-x (2018).

19 Wilm, A. *et al.* LoFreq: a sequence-quality aware, ultra-sensitive variant caller for uncovering cell-population heterogeneity from high-throughput sequencing datasets. *Nucleic Acids Res* **40**, 11189-11201, doi:10.1093/nar/gks918 (2012).

20 Benjamin, D. *et al.* (bioRxiv, 2019).

21 Chen, S., Zhou, Y., Chen, Y. & Gu, J. fastp: an ultra-fast all-in-one FASTQ preprocessor. *Bioinformatics* **34**, i884-i890, doi:10.1093/bioinformatics/bty560 (2018).

22 Langmead, B. & Salzberg, S. L. Fast gapped-read alignment with Bowtie 2. *Nat Methods* **9**, 357-359, doi:10.1038/nmeth.1923 (2012).

23 Li, H. *et al.* The Sequence Alignment/Map format and SAMtools. *Bioinformatics* **25**, 2078-2079, doi:10.1093/bioinformatics/btp352 (2009).

24 Tarasov, A., Vilella, A. J., Cuppen, E., Nijman, I. J. & Prins, P. Sambamba: fast processing of NGS alignment formats. *Bioinformatics* **31**, 2032-2034, doi:10.1093/bioinformatics/btv098 (2015).

25 Quinlan, A. R. & Hall, I. M. BEDTools: a flexible suite of utilities for comparing genomic features. *Bioinformatics* **26**, 841-842, doi:10.1093/bioinformatics/btq033 (2010).

26 Ramirez, F. *et al.* deepTools2: a next generation web server for deep-sequencing data analysis. *Nucleic Acids Res* **44**, W160-165, doi:10.1093/nar/gkw257 (2016).

27 Robinson, J. T. *et al.* Integrative genomics viewer. *Nat Biotechnol* **29**, 24-26, doi:10.1038/nbt.1754 (2011).

28 Zhang, Y. *et al.* Model-based analysis of ChIP-Seq (MACS). *Genome Biol* **9**, R137, doi:10.1186/gb-2008-9-9-r137 (2008).

29 Heinz, S. *et al.* Simple combinations of lineage-determining transcription factors prime cis-regulatory elements required for macrophage and B cell identities. *Molecular cell* **38**, 576-589, doi:10.1016/j.molcel.2010.05.004 (2010).

30 Jain, N. *et al.* Targeting phosphatidylinositol 3 kinase-beta and -delta for Bruton tyrosine kinase resistance in diffuse large B-cell lymphoma. *Blood Adv* **4**, 4382-4392, doi:10.1182/bloodadvances.2020001685 (2020).

31 Sehgal, L. *et al.* 14-3-3gamma-Mediated transport of plakoglobin to the cell border is required for the initiation of desmosome assembly in vitro and in vivo. *J Cell Sci* **127**, 2174-2188, doi:10.1242/jcs.125807 (2014).

**Figure S1**


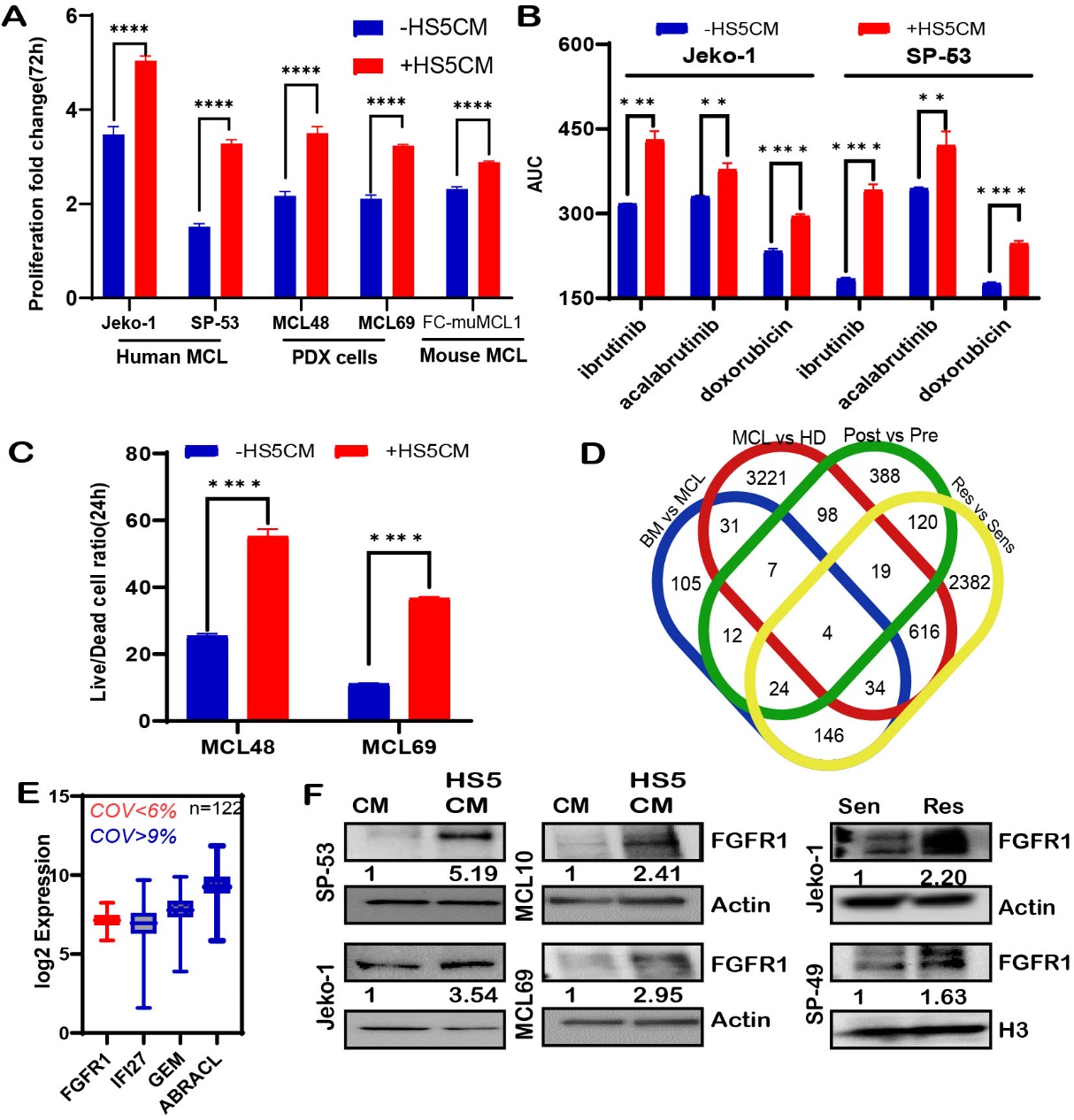


**Figure S2**


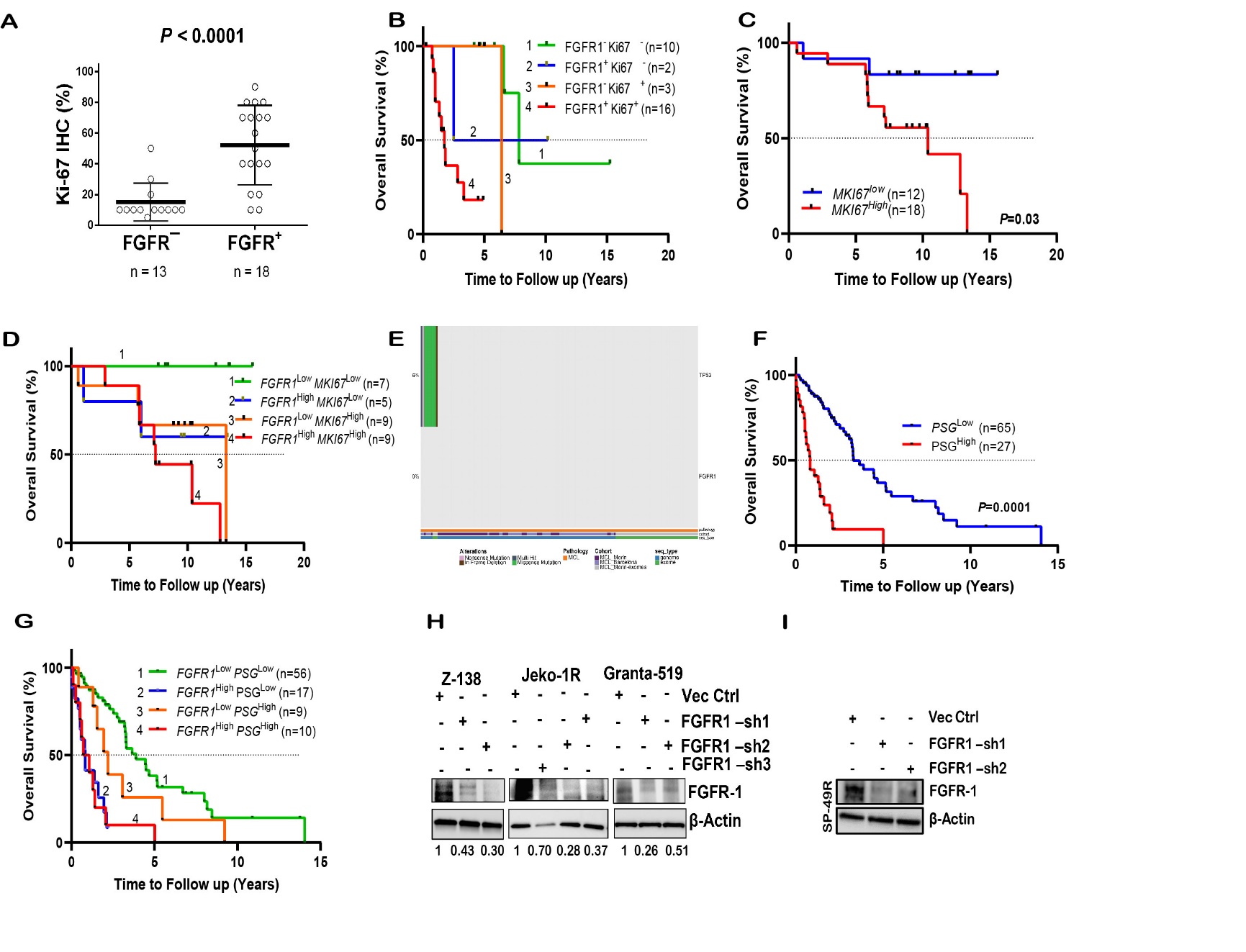


**Figure S3**


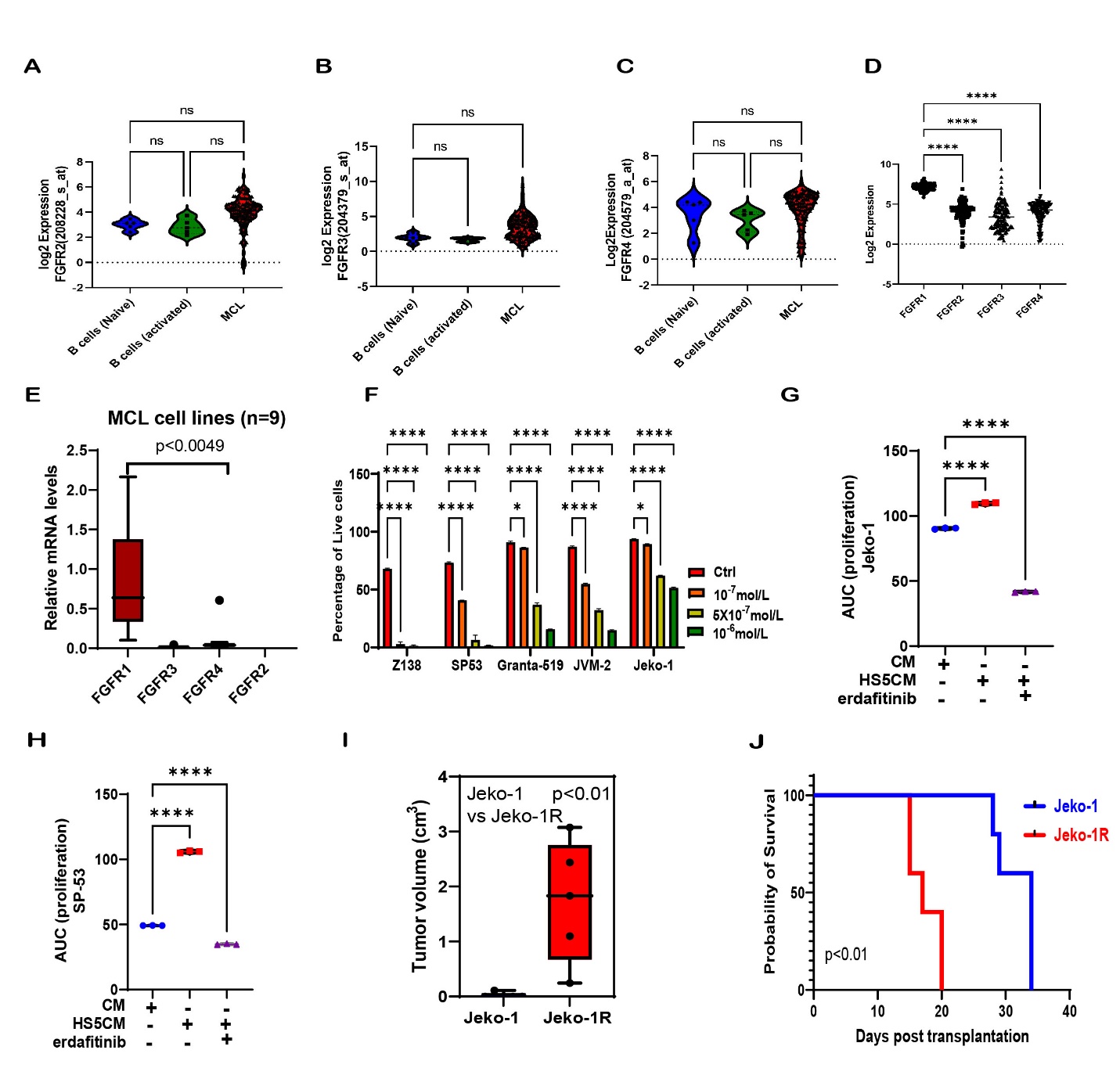


**Figure S4**


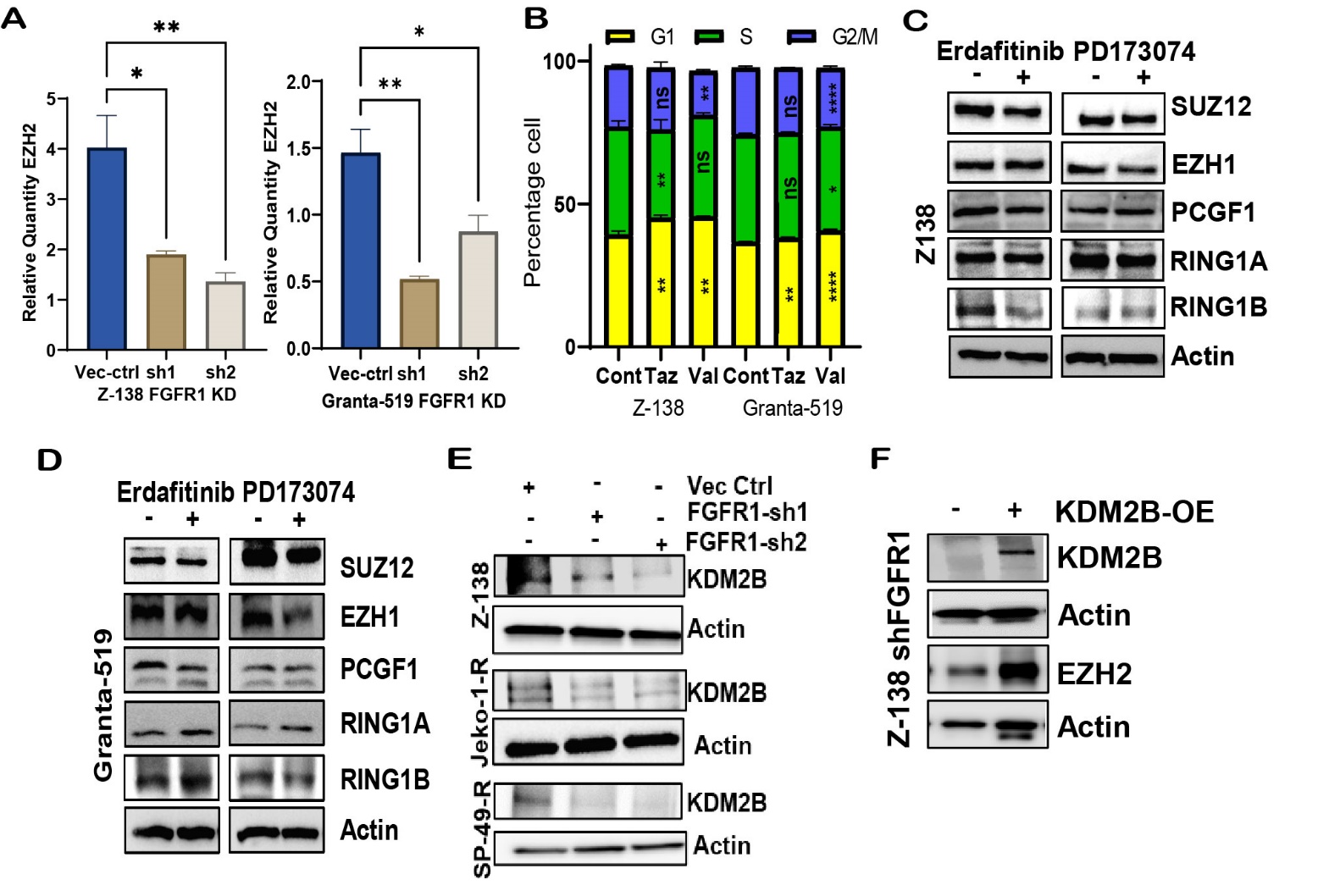


**Figure S5**


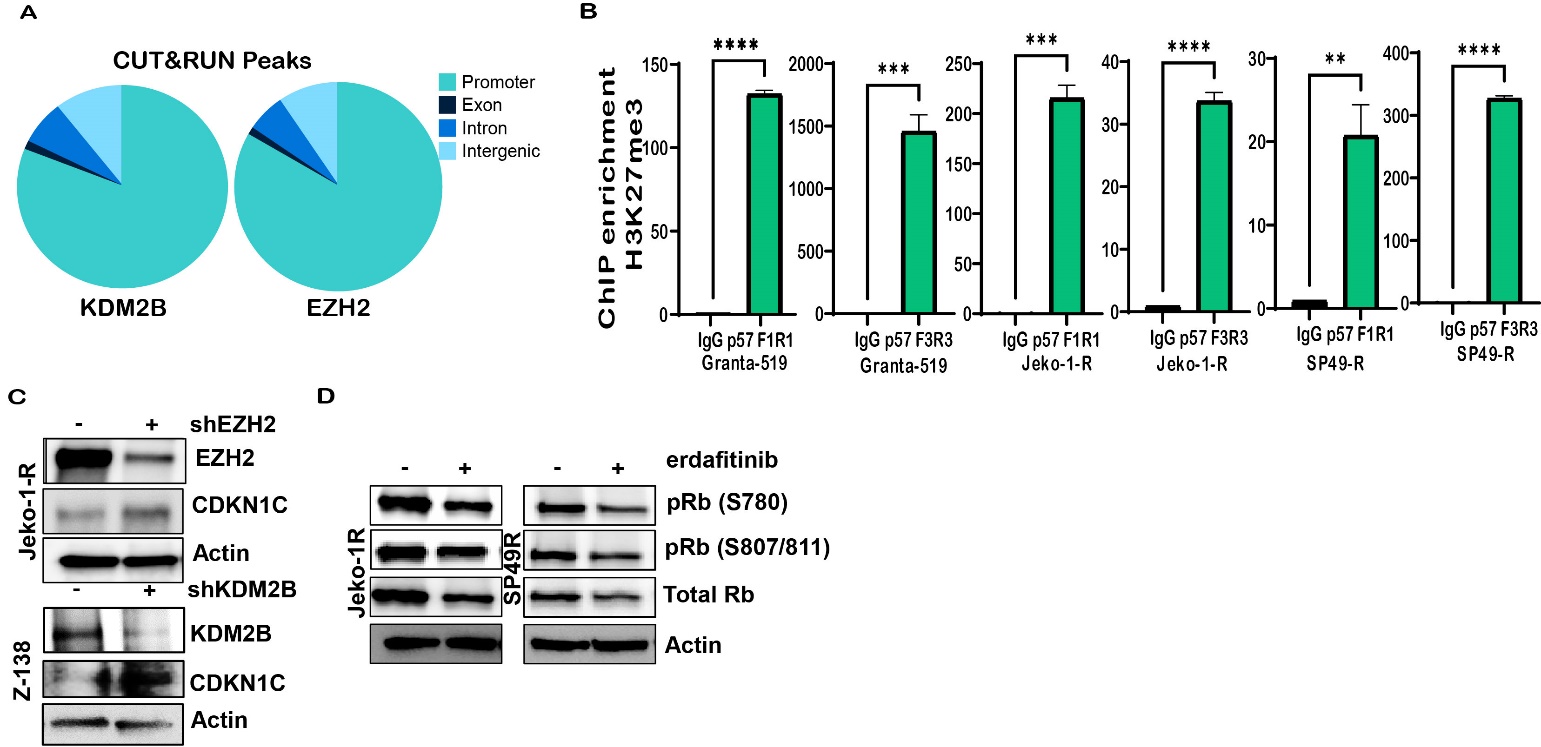


**Figure S6**


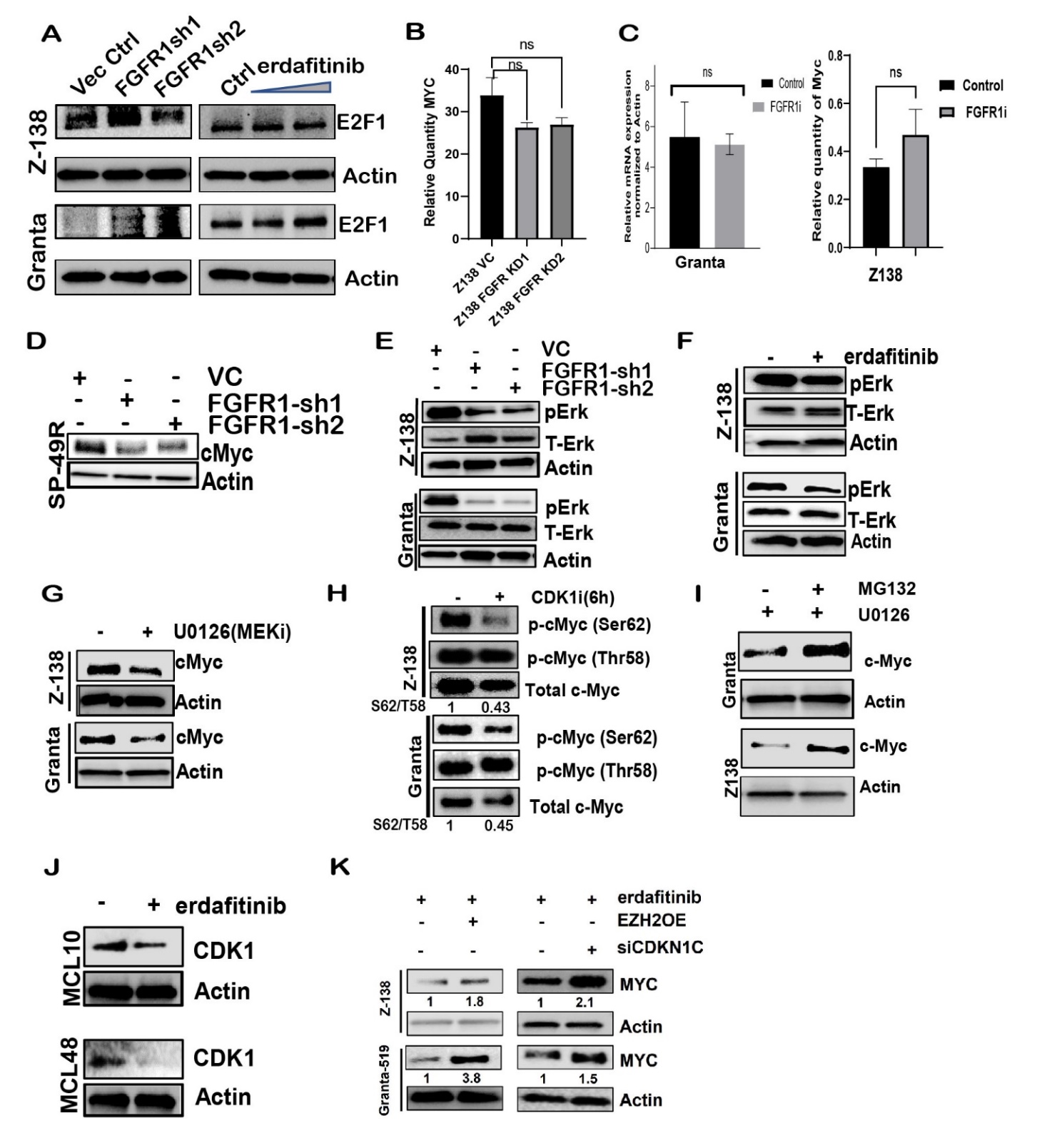


**Figure S7**


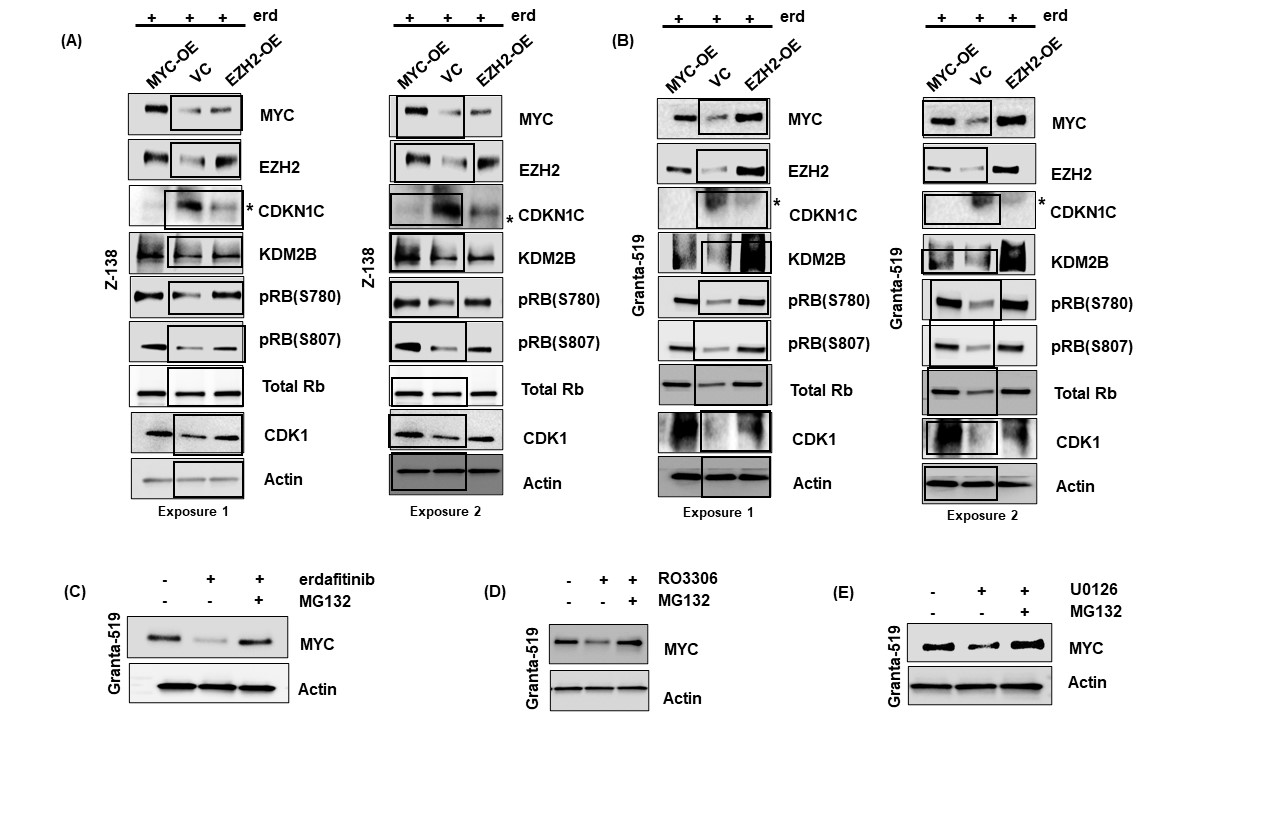

Supplement: Supplementary file 1 — Supplementary methods and Figures [file 41375_2023_2006_MOESM1_ESM.docx]
